# Indication of the sensitivity of Pinaceae species growing in Eastern Central Europe to ground-level ozone pollution

**DOI:** 10.1007/s11356-025-35905-7

**Published:** 2025-01-14

**Authors:** Veronika Lukasová, Svetlana Varšová, Lucia Žatková, Katarína Adamčíková, Anna Buchholcerová, Milan Onderka, Rastislav Milovský, Dušan Bilčík, Veronika Mináriková

**Affiliations:** 1 Earth Science Institute, Slovak Academy of Sciences, Dúbravská Cesta 9, Bratislava, 840 05 Slovakia; 2 Earth Science Institute, Slovak Academy of Sciences, Ďumbierska 1, Banská Bystrica, 974 11 Slovakia; 3 Institute of Forest Ecology, Department of Plant Pathology, and Mycology, Slovak Academy of Sciences, Akademická 2, Nitra, 949 01 Slovakia; 4 Slovak Hydrometeorological Institute, Jeséniova 17, Bratislava, 833 15 Slovakia

**Keywords:** O_3_ stress, Oxidative stability, Critical level, Conifers, Alpine zone

## Abstract

This study focused on testing the response of the assimilation apparatus of evergreen Pinaceae species to increasing levels of oxidative stress simulated in manipulative experiments. Needles were collected from mature individuals of *Pinus mugo*, *Pinus cembra*, *Pinus sylvestris*, *Abies alba*, and *Picea abies* at the foothill (FH) and alpine treeline ecotone (ATE) in the High Tatras (Western Carpathians). The injury index (INX), quantified by the modified electrolyte leakage (EL) method, indicated severe needle damage due to exposure to extremely high levels of O_3_. Ozonation induced changes in the chemical composition of the needles, which were detected via gas chromatography-mass spectrometry. The oxidative stability (OxS) indicator derived from INXs was used to determine the stomatal O_3_ flux-based critical level CL(OxS), with the threshold value of OxS at -0.05, corresponding to 5% injury to the needles. Assessment of the phytotoxic ozone dose (POD0) under ambient O_3_ and field environmental conditions during the 2023 growing season via CL(OxS) revealed that the studied species utilised between 18% (*Abies alba* FH) and 33% (*Pinus mugo* ATE) of their O_3_ tolerance potential. These results support our hypothesis that Pinaceae species growing in the High Tatras, which are part of the Alpine biogeographical region of Eastern Central Europe, are vulnerable to O_3_ concentrations significantly higher than the typical ambient O_3_ level in the natural environment.

**Supplementary Information:**

The online version contains supplementary material available at 10.1007/s11356-025-35905-7.

## Introduction

Vegetation in European mountain ranges is affected by various biotic and abiotic stressors (Badea et al. 2004; Mezei et al. 2017; Fernandez et al. 2021; Lukasová et al. 2021; Petrík et al. 2024), including air pollution by tropospheric ozone (O_3_) (Braun et al. 2017a, b; Hůnová et al. 2019; Janík et al. 2023). Tropospheric O_3_ is currently recognised as a widespread secondary pollutant that can inhibit plant life functions, particularly by suppressing vegetation vitality and reducing biomass accumulation (Pellegrini et al. 2011; Saitanis et al. 2014; Ducker et al. 2018; Feng et al. 2019; Grulke and Heath 2019; Ramya et al. 2023). The effects of O_3_ on woody plants have been documented in various studies that focused on the occurrence of O_3_ symptoms in leaf tissue (Vollenweider et al. 2003) and analyses of biomass, growth, physiology, and biochemistry (Wittig et al. 2009), as well as net primary production (Juráň et al. 2018). Several studies have used O_3_ fumigation in free air (Matyssek et al. 2010) or applied epidemiological approaches (Braun et al. 2014; Sicard et al. 2016; Braun et al. 2017a, b) and neural network analysis (Savi et al. 2020) to investigate O_3_ impacts on vegetation under field conditions. These studies particularly highlighted the reduction in photosynthetic carbon assimilation due to O_3_ and the resulting changes in carbon allocation in trees and forest ecosystems.

The oxidative stress induced by O_3_ alters plant cell functions, with the primary target being the photosynthetic apparatus in mesophyll cells. O_3_ molecules entering plant tissues through stomata induce the formation of reactive oxygen species (ROS), which trigger toxic oxidative processes such as membrane lipid peroxidation, protein oxidation, and DNA and RNA damage (Mittler 2002). Several stress markers, including ROS, reactive carbonyl species such as malondialdehyde (MDA), and photosynthetic pigments, have been identified as indicators of oxidative stress in plant tissue. However, these markers are not suitable for discriminating the differential oxidative stress applied singularly (Pellegrini et al. 2021). Various environmental stressors, such as drought, salinity, extreme temperatures, or the presence of toxic substances, can also generate ROS, leading to damage to membrane integrity. This damage may be tracked by the extent of electrolyte leakage (EL) in plant tissues (Whitlow et al. 1992; Bajji et al. 2002; Kovaleski and Grossman 2021).

Monitoring plant responses to O_3_ is crucial for estimating reference levels at which oxidative stress can severely disturb the oxidative balance of plants. The evidence of the detrimental effects of O_3_ on vegetation has led to the establishment of critical levels (CLs) for selected O_3_ metrics. The European Union’s legislation (Directive 2008/50/EC 2008) applies the exposure metric AOT40, which sets target values and long-term objectives for the protection of vegetation. However, the scientific community currently favours the O_3_ flux-based metric (POD_Y_—phytotoxic ozone dose above the *Y* threshold of stomatal O_3_ flux), which represents the uptake of O_3_ through the stomatal pores of leaves. Previous studies have suggested that the observed effects of O_3_ on vegetation are more strongly related to POD_Y_ than to AOT40 (Mills et al. 2011; Sicard et al. 2016; Paoletti et al. 2022). Both metrics consider threshold values below which plants can detoxify the effects of O_3_.

The detoxification capacity is species-specific and is accounted for by including an hourly cut-off Y flux in POD_Y_ and a concentration *X* in AOTX (with *X* being 40 ppb in AOT40) (CLRTAP 2017). The derivation of *Y* for long-lived organisms, such as forest trees, is based on modelling techniques using physiological data from experimental studies (Fuhrer 1994; Fuhrer et al. 1997). The epidemiological survey of POD_Y_ for adult forest trees, which compares *Y* thresholds, recommends the use of POD0 without a threshold limit (*Y* = 0) because it has both biological significance and practical utility (Sicard et al. 2016). Regardless of the metric adopted, several studies have pointed to the nonsignificant effect of elevated O_3_ or exceeded CL on vegetation status, which is often more significantly influenced by biotic or abiotic factors, nutritional status, climate, and site characteristics (Ferretti et al. 2018; Etzold et al. 2020; Lukasová et al. 2022; Bičárová et al. 2023).

The global concept of oxidative stress is defined as an imbalance between oxidants and antioxidants in favour of oxidants, leading to a disruption of redox signalling and control or resulting in molecular damage (Sies 2019). Different cell compartments have specific ROS generation and detoxification capacities and are vital for regulating ROS scavenging systems and keeping ROS below the threshold level to protect cellular components or initiate a signalling cascade (Hasanuzzaman et al. 2020). As a natural defence reaction in forest trees, the phenylpropanoid pathway, which leads to the accumulation of polyphenolic compounds (flavonoids, lignins, Dizengremel 2001) and other secondary metabolites with antioxidant capabilities, can be stimulated (Tonelli et al. 2015; Pellegrini et al. 2019). Similarly, enzymatic antioxidants such as ascorbate peroxidase, catalase, peroxidase, glutathione reductase, and superoxide dismutase (SOD) have been shown to significantly stimulate O_3_ stress to neutralise ROS (Paoletti et al. 2008; Castagna and Ranieri 2009; Bortolin et al. 2014; Picchi et al. 2017). The ability of plant tissues to mobilise enzymatic defence against uncontrolled oxidation may be a key factor in their tolerance to O_3_. Plant species and cultivars exhibit varying levels of sensitivity to O_3_, which is determined by heritable characteristics that influence biochemical and molecular processes related to O_3_ injury. However, their exact makeup remains unclear (Fiscus et al. 2005). The sensitivity of trees to O_3_ depends on various factors, such as the species; local, regional, and time scales of tree response to O_3_; differences in the ages of trees; spatial and temporal distributions of pollutants and precursors; and interactions with other biotic and abiotic stresses (Dizengremel 2001).

The manipulative experiment presented in this study builds on previous research from the High Tatra Mountains, which indicated the low sensitivity of *P. mugo* and *P. cembra* to oxidative stress with a rare occurrence of visible O_3_ injury signs on needle surfaces (Bičárová et al. 2023) and significant correlations between the greenness of *P. mugo* and climate variables compared with flux-based O_3_ doses in O_3_-rich mountain environments (Lukasová et al. 2021, 2022). This research was performed in the alpine treeline ecotone (ATE) zone of Tatra National Park, where the legally protected *Pinus cembra* L. naturally grows.

The primary goal of this study was to test the response of the assimilation apparatus of adult trees representing species of the Pinaceae family (*Pinus mugo* Turra, *Pinus cembra* L., *Pinus sylvestris* L., *Abies alba* Mill. and *Picea abies* [L.] Karst.) to gradually increasing oxidative stress simulated under laboratory conditions. The study hypothesised that these tree species, which grow in the High Tatras mountain environment, are vulnerable to O_3_ concentrations significantly higher than typical ambient O_3_ levels. The study focused on several specific objectives.

1. Indication of the oxidative stability (OxS) of the assimilation apparatus using the modified electrolyte leakage method to test the direct effect of O_3_ without the influence of other environmental factors. Differences in OxS among Pinaceae species growing in the O_3_-rich environments of the alpine treeline ecotone (ATE) and foothill (FH) positions, which have relatively low average seasonal O_3_ concentrations, were analysed.
2. Changes in the chemical composition of the sampled plant material exposed to ambient O_3_ under field conditions and to gradually increasing ozonation under laboratory conditions were detected via gas chromatography-mass spectrometry (GC–MS).
3. We proposed O_3_ flux-based critical levels against oxidative stability (CL(OxS) for the POD0 metric for each tree species and compared them with the actual O_3_ doses derived from the ambient air O_3_ levels.

## Materials and methods

### Sampling design

**Fig. 1:**
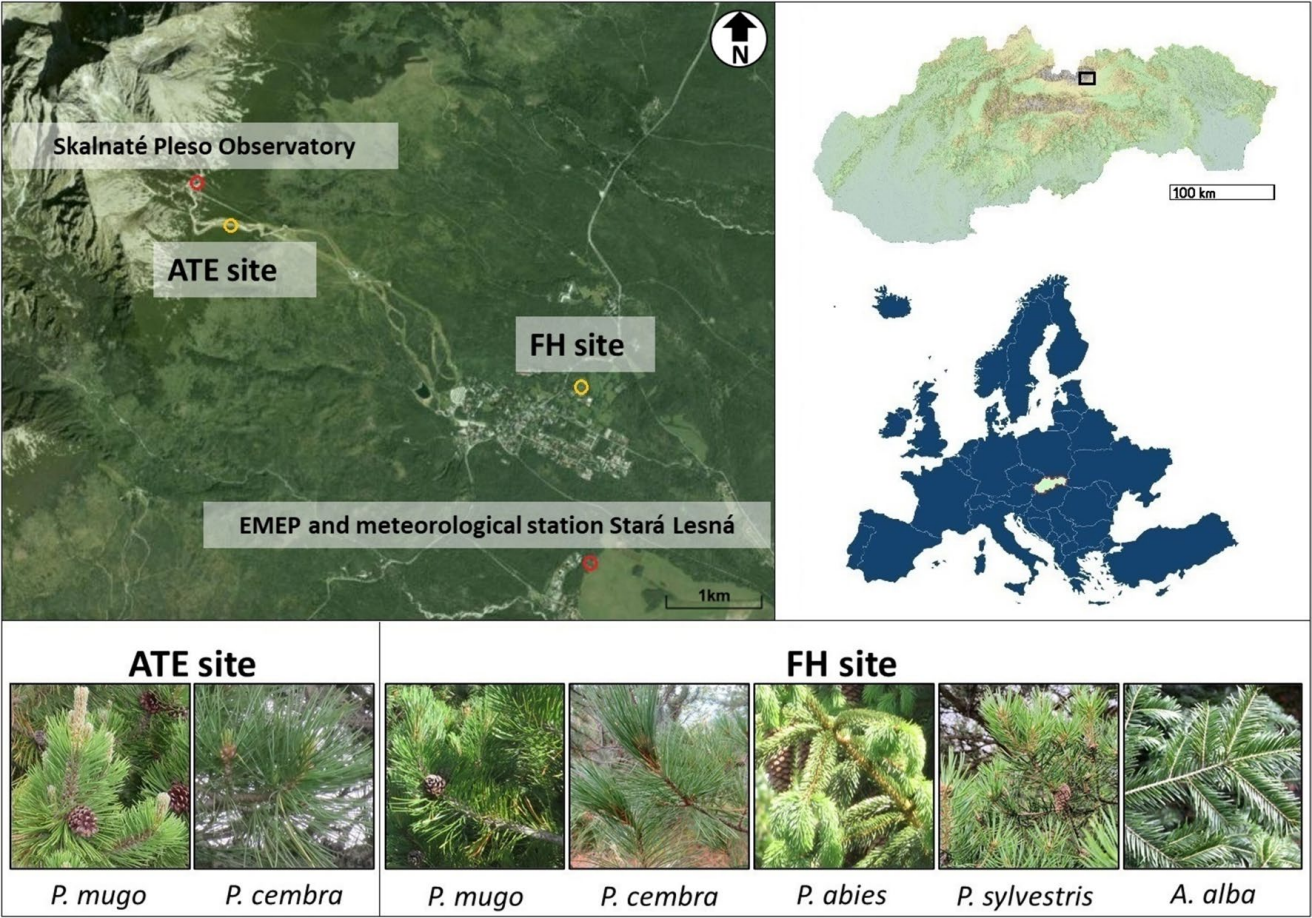
Sites for collecting needle samples from adult individuals of the Pinaceae species at the foothill (FH) in the Exposition of Tatra Nature, Botanical Garden in Tatranská Lomnica, and at the alpine treeline ecotone (ATE) in the High Tatra Mts. The study region is marked with a rectangle on the map of Slovakia, Eastern Central Europe

Experimental material was collected from selected adult individuals representing native Pinaceae species growing in forested areas of the High Tatra Mountains (Slovakia, Eastern Central Europe). High Tatras are part of the Alpine biogeographical region (Cervellini et al. 2020) within Western Carpathians. Needle samples were obtained from two zones (Fig. 1) with different altitudes and average ambient O_3_ concentrations: (i) the foothill zone (FH, 49° 10′ 07″ N; 20° 17′ 19″ E, 830 m a.s.l.) and (ii) the alpine treeline ecotone zone (ATE, 49° 11′ 02″ N; 20° 14′ 16″ E, 1600 m a.s.l.).

At FH, samples of *Pinus mugo* Turra, *Pinus cembra* L., *Pinus sylvestris* L., *Abies alba* Mill., and *Picea abies* [L.] Karst. were collected at the Botanical Garden—Exposition of Tatra Nature in Tatranská Lomnica. Measurement of the O_3_ concentration at the nearest O_3_ monitoring station, EMEP (European Monitoring and Evaluation Programme), in Stará Lesná during the period 2000–2020 (EBAS database (https://ebas-data.nilu.no/), revealed that the level of the seasonal O_3_ concentration at the FH varies from ~ 33 ppb during the summer‒autumn season from June to September to ~ 36 ppb during the spring‒autumn season from April to September. For the same period, seasonal spring‒autumn O_3_ concentrations reached a substantially higher level of ~ 52 ppb at ATE (Lukasová et al. 2022). At ATE, only *P. mugo* and *P. cembra* were available to be sampled (Fig. 1) because climate and environmental conditions limit the growth of other Pinaceae species at these high altitudes.

Field sampling was conducted at the end of the growing season (GS) in early October 2023. At both sites, three adult individuals of each species that presented no signs of disturbance were selected for needle sampling. Needles were collected from the outer side of the crown from three different branches, with a focus on 2-year-old (C + 2) needles where O_3_ injury is most pronounced (Bičárová et al. 2019). The needles were carefully pulled, wrapped in aluminium foil, and placed in a plastic zip-lock bag. The needle samples were processed upon arrival at the laboratory. Initially, the needles were cut into 1 cm pieces, with the bases and tips removed. Then, for each species and zone, samples of 6 × 200 mg were prepared for the EL method, and approximately 3 × 5000 mg were prepared for the GC–MS procedure.

### Modified electrolyte leakage method

The electrolyte leakage (EL) method is commonly used to assess plant responses to stress conditions directly proportional to cell membrane damage. This method was modified for our study to include artificial ozonation of plant samples in a laboratory chamber under controlled O_3_ concentrations (Bičárová et al. 2023). Previous studies have indicated that *P. mugo* and *P. cembra* exhibit low sensitivity to ambient O_3_ concentrations (Lukasová et al. 2022; Bičárová et al. 2023). Therefore, in addition to needles affected by O_3_ in the natural environment, we analysed needle samples exposed to extremely high artificial O_3_ concentrations (≈ 150 ppm) with gradually prolonged exposure times (1 h, 3 h, 5 h, 7 h, and 10 h). The value of 150 ppm represents the accumulated value of the average hourly O_3_ concentration that affects coniferous trees during the growing season in the natural environment from June to September. The value of 150 ppm was derived from a simple calculation: 52 ppb × 24 h of day × (30 + 31 + 31 + 30) days of the growing season = 150 ppm (after being rounded on tenths). Samples of 200 mg in weight intended for artificial ozonation were placed in perforated 1.5-ml Eppendorf tubes, which were inserted into special holders to ensure a uniform flow of ozonised air in the chamber. During ozonation, O_3_ penetrated the cellular structures in two ways: through the stomatal pores, which were not closed air tight after the sampling manipulation, and through the cross-sectional profiles created by cutting, which allowed O_3_ to diffuse into the interior spongy mesophyll area. Ozonation was conducted in five series for each species and location. The samples were placed in the laboratory chamber simultaneously and then removed gradually at different intervals. During ozonation, the samples were exposed to progressively increasing O_3_ doses, which were calculated as the sum of the hourly O_3_ concentrations over the defined exposure period (sumO_3_). The exposure times with the corresponding sumO_3_ values are presented in Table 1. The samples collected at ATE were subjected to sumO_3_ ranging from 154 to 2152 ppm, whereas the cumulative hourly O_3_ concentration measured over the GS 2023 in the natural environment was ~ 154 ppm. The samples from the FH zone were exposed to sumO_3_ ranging from 101 to 1764 ppm, with the cumulative hourly O_3_ concentration in ambient air being ~ 101 ppm. Artificial ozonation was performed in a fluid laboratory chamber with an O_3_ input flow controlled by an O_3_ device, specifically the Thermo Electron Environmental 49C. Extremely high artificial O_3_ concentrations were generated electrochemically via Koizon 300 equipment, a product of Gemke Technik GmbH, with the flow rate regulation set at 0.5 Lpm. To confirm the presence of O_3_, passive indigo filters were placed inside the chamber, and an aqua indigo solution was used at the chamber's output. The qualitative test of O_3_ presence was based on a chemical reaction between indigo carmine and ozone, resulting in a colour change from dark blue to light yellow (Maruo et al. 2009; Varšová et al. 2024).

**Table 1:** Sum of the hourly O_3_ concentrations (sumO_3_(n), ppm) to which samples collected in the field at different altitudes (ATE, FH) were exposed

Zone of sample collection	SumO_3_(*n*) in ppm for a series of samples					
	1	2	3	4	5	6
ATE	154	323	668	991	1312	1825
FH	101	267	600	932	1265	1764
Exposure time (h)	:	1	3	5	7	10

SumO_3_(1) represents the sum of the hourly concentrations of ambient O_3_ during the 2023 growing season; sumO_3_(2–6) indicates the total dose of additional artificial ozonation lasting from 1 to10 h

After ozonation, each sample, including the non-ozonised samples, underwent the EL procedure to determine the injury index, INX(*n*), where (*n*) represents the serial number of samples from 1 to 6. INX(*n*) was derived from measurements of the EL conductivity of ultrapure water and solutions with needles before and after the destruction of the cell membranes in an autoclave (121 °C, 20 min). EL conductivity was measured by a calibrated conductometer TDS Testr 11 (Eutech Instrument, Thermo Fisher Scientific Inc., Singapore). The entire procedure is described in detail in Bičárová et al. (2023). The INX(1) was associated with samples not exposed to artificial O_3_ and describes the response to ambient O_3_ monitored by automatic O_3_ devices at ground-based monitoring stations. INX(2–6) reflect the injury related to the effect of controlled artificial O_3_ exposure under additional graduated ozonation in the laboratory setting according to Table 1. The values of INX(1–6) were used to model INX(0), which substituted the control samples representing conditions without O_3_ pollution.

The oxidative stability of needles as a measure of cell membrane destruction was then calculated as the difference between INX(*n*) and INX(0), with the result converted from a percentage to a decimal format. Therefore, OxS ranged from − 1 to 0, where an OxS equal to 0 indicated that the plant tissue was oxidatively stable, indicating that no oxidative effect was detected. An OxS value less than 0 indicates the degree of plant tissue instability or sensitivity to oxidative stress. An OxS value of -0.05 was suggested as a threshold, with exceedance indicating a 5% injury to plant tissues caused by O_3_. On the basis of the fitting curve from the scatterplot relationship between OxS and sumO_3_, the sumO_3_ and avgO_3_ values corresponding to this 5% threshold were derived.

### Mass spectrometry and chromatography analyses

The GC–MS technique, which uses devices such as a Trace GC Ultra gas chromatograph coupled with an ITQ 900 ion trap mass spectrometer (Thermo Scientific), was applied to track changes in the chemical composition of needle samples of Pinaceae species exposed to ambient air and artificial ozonation but not processed via the EL method. A quantitative and qualitative analysis of the total lipid extract (TLE) from dried needle samples was conducted. The chemical components were separated on a nonpolar capillary column ZB5 (Phenomenex) at temperatures ranging from 60 to 320 °C with a gradient of 4 °C min^–1^.

The components were identified through mass spectral matching with the National Institute of Standards and Technology (NIST) library and confirmed by comparing retention times. Terpenoids were specifically identified via selected ion monitoring of characteristic fragments and quantified via total-ion-current (TIC) chromatograms (Žatková et al. 2023). The peak area of each detected molecule was normalised to the total peak area of all identified molecules, and the results were expressed as percentages. TLE was obtained from dried needle samples via Soxhlet extraction with a solvent mixture of 90% dichloromethane and 10% methanol (Bechtel et al. 2018). This procedure was applied to three series of 5000 mg samples exposed to sumO_3_(*n*) for series 1, 3, and 6 (Table 1), which included samples reflecting the effect of the ambient O_3_ concentration over the GS 2023 and samples exposed to artificial ozonation for 3 and 10 h.

### Modelling ozone doses and O_3_ flux-based critical levels

Ozone doses were modelled via the DO_3_SE model (Emberson et al. 2000; Büker et al. 2015; SEI 2023), which estimates the stomatal deposition (or stomatal flux, Fst) of O_3_ for selected European land cover types and plant species. The DO_3_SE model parameterisation (Table S1) according to the built-in preset for Boreal-Coniferous forests (CLRTAP 2017), with modifications of selected parameters for *P. mugo* (Bičárová et al. 2019) and *P. cembra* (Buchholcerová et al. 2021), was employed. For *P. sylvestris* and *A. alba*, parameterisation was based on related species, specifically *P. cembra* and *P. abies*, respectively.

The DO_3_SE model processed O_3_ and meteorological input data (Table S2) measured at Stará Lesná, near the FH zone, which is part of the European Air Quality Monitoring Network (EMEP 2020) and Skalnaté Pleso Observatory at ATE provided by the Earth Science Institute of the Slovak Academy of Sciences. The O_3_ concentrations were monitored via UV absorption O_3_ analysers—Teledyne API T400 at the FH location and Thermo Electron Environmental 49C at the ATE location. Meteorological variables were recorded by a reliable measurement system based on a PROlog ultralow-power datalogger (Physicus, SK) in both zones.

The model output for stomatal O_3_ flux (Fst) is aggregated to the metric POD0, which is the phytotoxic ozone dose POD_Y_ without threshold limitation (*Y* = 0). This approach was used because of incomplete data on the detoxification for all the analysed species. POD is expressed in mmol m^−2^ per projected leaf area (PLA); in this study, we used the shortened form mmol m^−2^. The use of POD0 was also recommended by Sicard et al. (2016).

Two series of POD0 model calculations were performed: (i) POD0 uptake by individual species in the natural environment (FH, ATE) during the GS 2023 using hourly O_3_ concentrations monitored by O_3_ analysers under field conditions and (ii) POD0 projected for critical levels against oxidative stability (CL(OxS)) considering seasonal avgO_3_ (ppb) related to OxS at the threshold level of -0.05. The average hourly O_3_ concentration was calculated by dividing sumO_3_ by the number of hours in the GS 2023. The GS included months where no days had all-day hourly air temperatures below 5 °C. Accordingly, the GS in the FH zone lasted from May 1st to September 30th, whereas in the ATE zone, it started on June 1st and ended on September 30th (Fig. S1).

## Results

### Injury to cell membrane integrity

The INX(*n*) values (Table 2) revealed a progressive increase in ozonation-induced injury across all the analysed conifer species as the sumO_3_ gradually increased (Table 1). The greatest degree of injury caused by environmental stress under ambient O_3_ conditions (INX(1)) was observed in *P. abies* at 31.0%. This injury increased to 58.4% after artificial ozonation (INX(6)), which was two to three times greater than that observed in other species and locations (Table 2). However, *P. abies* and *A. alba* presented the smallest increase in INX(*n*) values when the INX(1) values were compared with the final INX(6) values after exposure to artificial ozonation. When the INX(1) values of *P. mugo* and *P. cembra* were compared, both species presented greater injuries at ATE than at FH. Among the pine species, *P. cembra* ATE had the highest INX(*n*) in the series of INX(1) to INX(4), but in the later ozonation series, INX(5) and INX(6) were surpassed by both *P. mugo* FH and ATE (Table 2).

**Table 2:** Summary of the effects of ozonation on samples, expressed as injury indices INX(*n*). INX(1) represents the response to the cumulative ambient O_3_ concentration (sumO_3_) recorded in different zones (ATE, FH) during the 2023 growing season

Zone	Pinaceae species	INX(*n*) in % for a series of samples						
		0	1	2	3	4	5	6
ATE	*Pinus mugo*	11.0	12.7	14.3	17.1	23.7	34.9	43.2
	*Pinus cembra*	14.8	16.4	16.6	24.4	25.0	33.4	42.8
FH	*Pinus mugo*	9.3	10.1	13.2	14.9	21.5	38.9	48.4
	*Pinus cembra*	9.1	10.7	11.8	11.5	21.3	25.6	38.3
	*Pinus sylvestris*	12.1	12.4	15.4	17.5	24.0	25.8	40.4
	*Abies alba*	10.0	10.0	11.9	16.0	20.9	19.2	32.0
	*Picea abies*	28.6	31.0	30.9	36.6	38.4	41.5	58.4

**Fig. 2:**
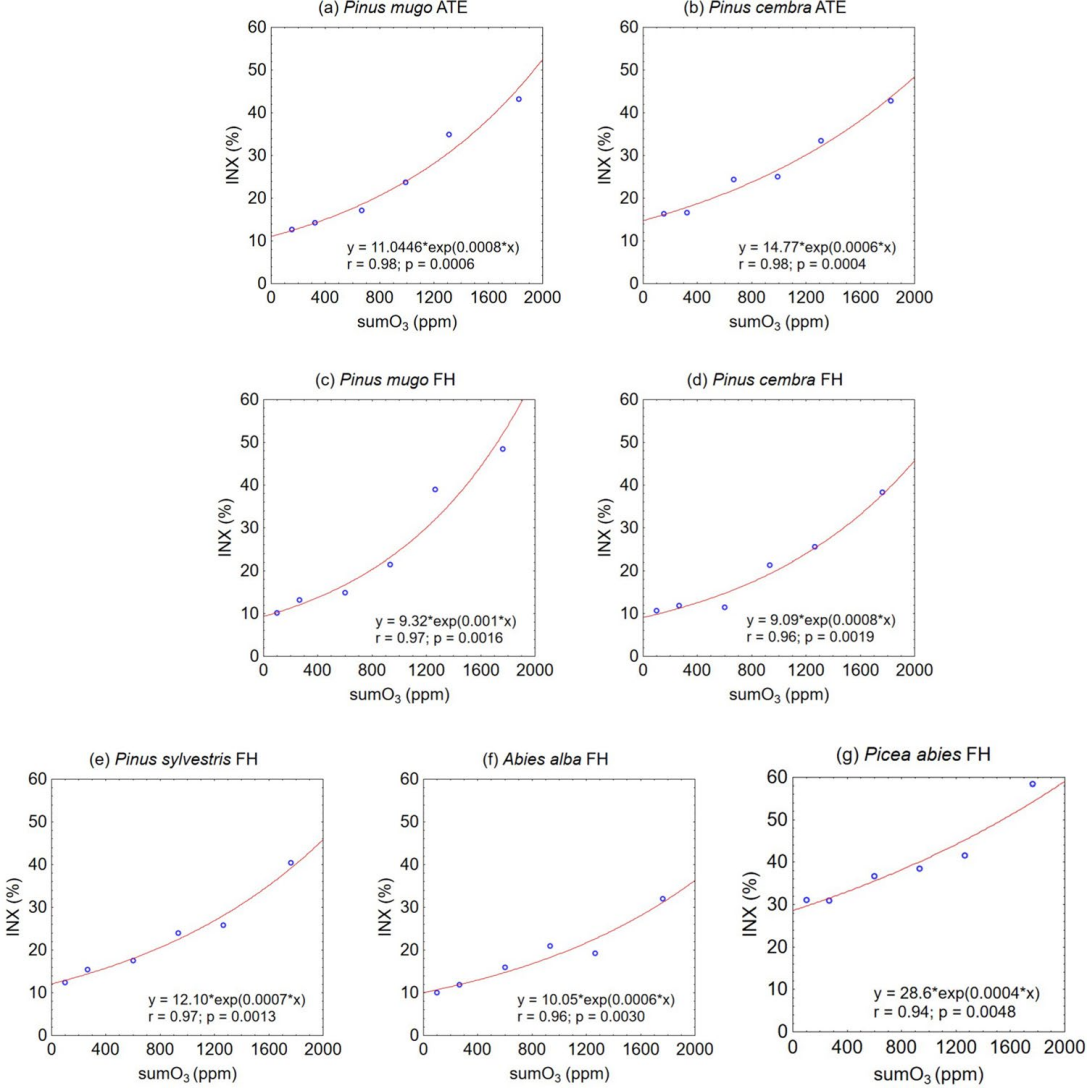
Scatter plots showing injury indices (INX(*n*)) derived from increasing O_3_ concentrations fitted with exponential functions to predict the value INX(0) for sumO_3_ = 0 ppm, which represents the sensitivity of Pinaceae species to the direct effect of O_3_, eliminating the influence of other environmental factors for each species separately

INX(2–6) reflect the effects of controlled O_3_ exposure, as shown in Table 1. INX(0) estimates the injury effect at zero cumulative O_3_ exposure (sumO_3_ = 0 ppm) modelled by the exponential functions shown in Fig. 2

The relationships between INX(*n*) and sumO_3_ for *n* = 1–6 were fitted with an exponential function to define the INX(0), i.e., the injury corresponding to sumO_3_ = 0 ppm for each species and location separately (Fig. 2). This INX(0) was used as a reference level in the calculation of oxidative stability. Compared with linear or polynomial models, the exponential function provided the highest correlation coefficient (*r*) and the lowest level of marginal significance (*p*).

### Changes in the chemical composition of the needle samples after ozonation

**Fig. 3:**
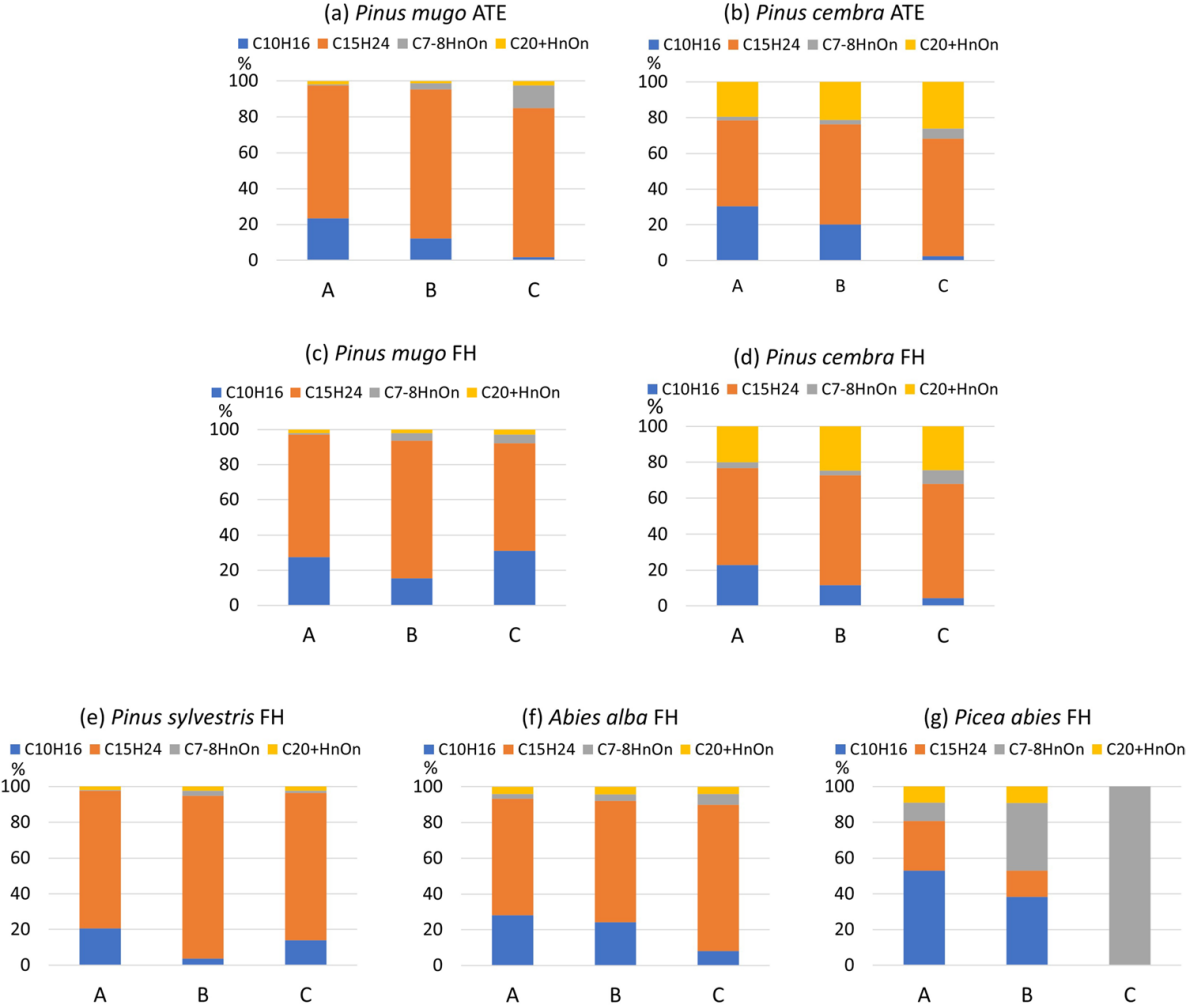
Changes in the chemical composition of samples identified by the GC–MS analytical method—percentages of monoterpenes C_10_H_16_, sesquiterpenes C_15_H_24_, and two groups of hydrocarbon oxidation products C_7–8_H_n_O_n_ and C_20+_H_n_O_n_ for individual species and zones between categories of samples exposed to (A) ambient O_3_ (series 1), (B) 3 h of artificial ozonation (series 3), and (C) 10 h of artificial ozonation (series 6)

The chemical components in the lipid extracts of the needle samples identified via the GC–MS technique were classified into four main groups (Table 3): monoterpenes (C_10_H_16_), sesquiterpenes (C_15_H_24_), and two groups of hydrocarbon oxidation products (C_7–8_H_n_O_x_ and C_20+_H_n_O_x_). These components changed in proportion after exposure to artificial ozonation. A detailed description of the identified chemical substances is provided in Table S3. The analyses revealed a similar chemical composition of needles among the species studied (Fig. 3; Fig. S3a–f), except for *P. abies.* In *P. abies*, the GC analyser detected a large, single group of substances, likely due to insufficient separation in the chromatograph column (Fig. 3g, Fig. S3g). As a result, *P. abies* could not be included in the chemical composition analyses with the other species.

**Table 3:** List of hydrocarbon compounds identified in needle samples by the GC‒MS technique sorted by increasing retention index (RI)

No	RI	Name	Formula
Monoterpenes			
I	1011	γ-terpinene	C_10_H_16_
II	1042	α-thujene	C_10_H_16_
III	1079	α-pinene	C_10_H_16_
Oxidation products			
IV	1213	Benzoic acid	C_7_H_6_O_2_
V	1246	Coumaran	C_8_H_8_O
Sesquiterpenes			
VI	1354	δ-elemene	C_15_H_24_
VII	1442	α-cubebene	C_15_H_24_
VIII	1506	Germacrene D	C_15_H_24_
IX	1537	γ-cadinene	C_15_H_24_
X	1544	δ-cadinene	C_15_H_24_
Oxidation products			
XI	2381	Dehydroabietic acid methyl ester	C_21_H_30_O_3_
XII	2845	Dehydroisoandrosterone acetate	C_21_H_30_O_3_
XIII	3271	α-tocopherol	C_29_H_50_O_2_

**Fig. 4:**
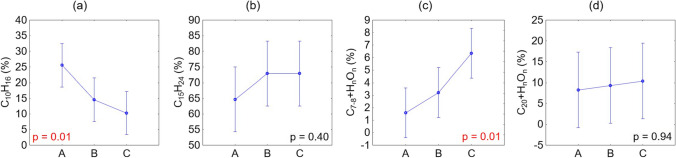
Results of the ANOVA analysis of the changes in the percentages of **a** monoterpenes, C_10_H_16_, **b** sesquiterpenes, C_15_H_24_, and two groups of hydrocarbon oxidation products, **c** C_7–8_H_n_O_n_, and **d** C_20+_H_n_O_n_, across all species and zones. The analysis compared groups of samples exposed to (A) ambient air O_3_ (series 1); (B) 3 h of artificial ozonation (series 3), and (C) 10 h of artificial ozonation (series 6). Significant differences between categories with *p* < 0.05 are indicated in red. The vertical bars denote 0.95 confidence intervals

The sesquiterpenes C_15_H_24_ dominated the samples from *P. mugo*, *P. cembra*, *P. sylvestris*, and *A. alba* (Fig. 3). When analysing changes in chemical composition across all species and zones between categories (A) ambient O_3_ (series 1), (B) 3 h of artificial ozonation (series 3), and (C) 10 h of artificial ozonation (series 6), ANOVA indicated no significant differences in the percentage of C_15_H_24_ (Fig. 4b). The consistently high percentages of C_15_H_24_ in the samples after ozonation suggested its robustness against oxidation.

In samples affected only by ambient O_3_, monoterpenes (C_10_H_16_) had the second-highest percentage across all samples (Fig. 3, Column A). After ozonation (Fig. 3, Columns B and C), the content of C_10_H_16_ decreased in all species except for *P. mugo* FH. Changes in chemical composition across all species and zones between categories A, B, and C were confirmed by ANOVA, which revealed significant differences in the percentage of C_10_H_16_ (Fig. 4a). The highest percentages of C_10_H_16_ were recorded in samples exposed to ambient O_3_, with a decrease observed as the sumO_3_ from artificial ozonation increased. Ozonation cleaves double bonds and results in the formation of various oxygenated compounds. The expected primary ozonation products included aldehydes and ketones. Further reactions could produce carboxylic acids, alcohols, and peroxides. However, in the ozonised samples, we did not detect aldehydes, ketones, or the presence of the malondialdehyde marker. MDA is a commonly known marker of oxidative stress, as it is one of the final products of polyunsaturated fatty acid peroxidation in cells. Instead, we observed a significant increase in the proportion of oxygenated derivatives of hydrocarbons in the cyclic C_7–8_ hydrocarbon oxidation product group (Fig. 4c). This group (C_7–8_H_n_O_n_) included compounds such as benzoic acid, benzofuran, and coumaran, with benzoic acid showing the most pronounced increase.

A group of oxygenated derivatives of diterpenes was dominant mainly in the *P. cembra* samples at both FH and ATE (Fig. 3) because of the contributions of dehydroabietic acid methyl ester and dehydroisoandrosterone acetate, whereas the percentage of α-tocopherol was negligible. Among the remaining species, only low percentages of this group were detected (Fig. 3). ANOVA indicated that this group of C_20+_H_n_O_n_ did not significantly react to increasing ozonation (Fig. 4d).

### Sensitivity of Pinaceae species to manipulative oxidative stress

Analyses of the oxidative stability of needle samples provided insight into species-specific sensitivity to ozone damage. As summarised in Table 4, OxS decreased with increasing levels of artificial ozonation. Under ambient O_3_ conditions, OxS(1) fluctuated around 0 for all Pinaceae species, indicating minimal disturbance from ground-level O_3_ on needles in the natural environment. The highest OxS was observed in *A. alba*, where the OxS decreased to −0.22 between series 1 and 6. The lowest OxS was found in *P. mugo* in both zones, with a decrease to − 0.39 in the ATE zone and − 0.32 in the FH zone in series 6.

**Table 4:** Oxidative stability of samples collected from Pinaceae species in different zones (ATE, FH) at the end of the 2023 growing season

Zone	Pinaceae species	OxS(*n*) for a series of samples						
		1	2	3	4	5	6	sumO_3_(OxS)
ATE	*Pinus mugo*	− 0.01	− 0.03	− 0.06	− 0.12	− 0.24	− 0.32	464.0
	*Pinus cembra*	− 0.01	− 0.02	− 0.09	− 0.10	− 0.18	− 0.28	511.3
FH	*Pinus mugo*	− 0.01	− 0.04	− 0.06	− 0.12	− 0.30	− 0.39	426.0
	*Pinus cembra*	− 0.02	− 0.03	− 0.02	− 0.12	− 0.17	− 0.29	518.2
	*Pinus sylvestris*	0.00	− 0.03	− 0.05	− 0.12	− 0.14	− 0.28	506.1
	*Picea abies*	− 0.02	− 0.02	− 0.08	− 0.10	− 0.13	− 0.30	455.8
	*Abies alba*	0.00	− 0.02	− 0.06	− 0.11	− 0.09	− 0.22	606.8

OxS(1) represents the response to the ambient cumulative O_3_ concentration (sumO_3_), whereas OxS(2–6) reflects the effects of controlled O_3_ exposure in a laboratory setting. SumO_3_(OxS) in ppm is the cumulative O_3_ concentration corresponding to OxS = -0.05 modelled via a polynomial regression function of OxS (Fig. 3) for each species and zone

**Fig. 5:**
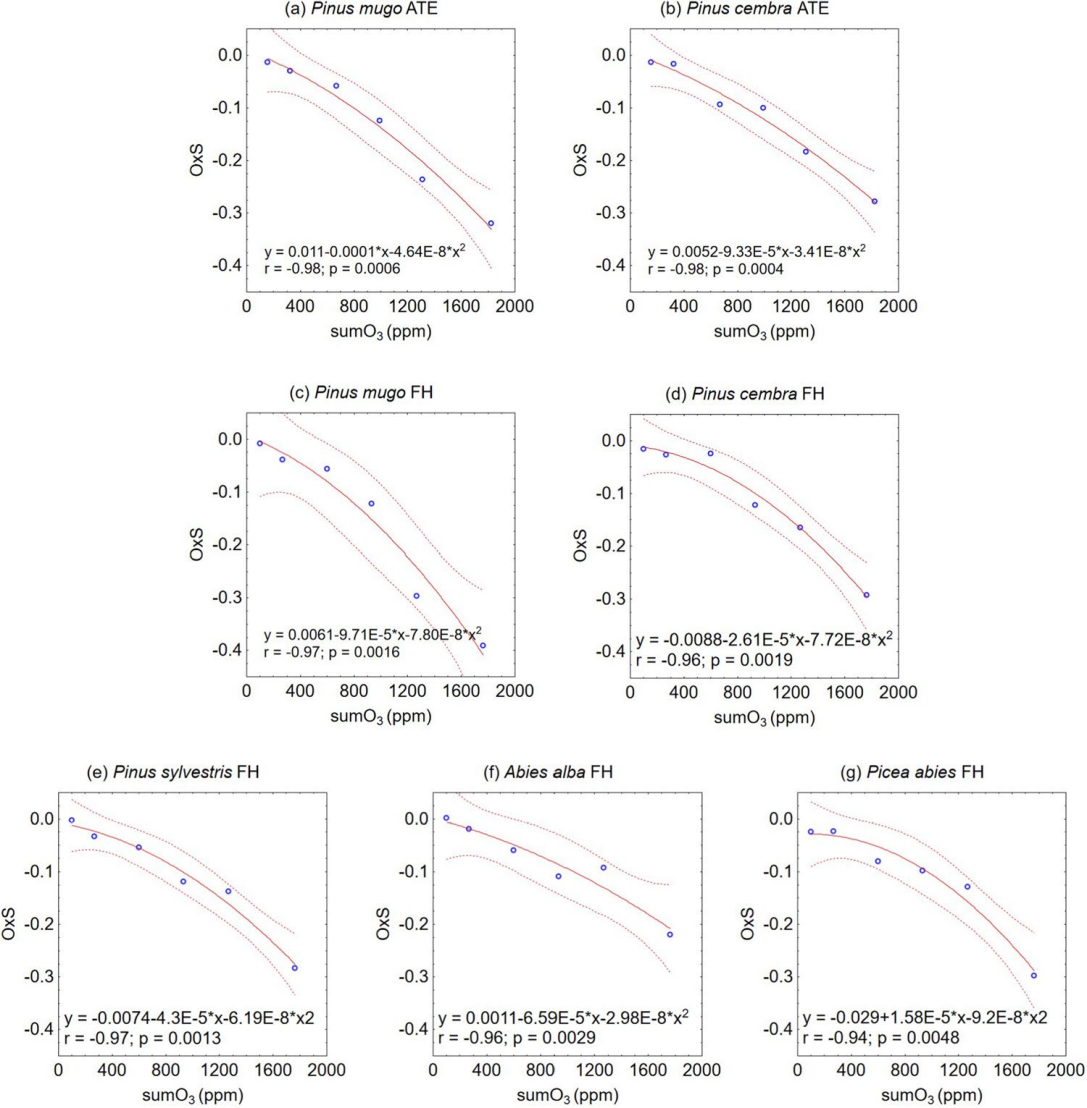
Polynomial regressions with 0.95 confidence intervals used to derive the sumO_3_(OxS) for the threshold value of OxS = -0.05, calculated separately for each species of Pinaceae

The relationships between OxS and increasing sumO_3_ were described by polynomial functions. Using these equations (Fig. 5), we derived the sumO_3_(OxS) corresponding to an OxS of -0.05, which indicates a 5% injury of needles caused by ozonation for each species and zone separately (Table 4). The lowest sumO_3_(OxS) concentrations predicted to induce 5% injury were 426.0 ppm and 464.0 ppm for *P. mugo* in the FH and ATE zones, respectively, and 455.8 ppm for *P. abies* FH. For *P. cembra*, the 5% injury was modelled at sumO_3_(OxS) values of 518.2 ppm and 511.3 ppm at the FH and ATE zones, respectively. A similar sumO_3_(OxS) value was identified for *P. sylvestris* FH (506.1 ppm). The highest sumO_3_(OxS) that could lead to injury of 5% was modelled for *A. alba*, which reached 606.8 ppm. The modelled sumO_3_(OxS) exceeded the sumO_3_(1) calculated from the measurements under ambient conditions, which were ~ 101 ppm at FH and ~ 154 ppm at ATE (Table 1), by 3–6 times, depending on the species and location.

### O_3_ flux-based critical levels for species of Pinaceae against OxS

The model results of the flux-based O_3_ dose included two model series of POD0 calculations considering (i) the ambient O_3_ concentration and (ii) the O_3_ concentration projected for the critical level against the OxS threshold of -0.05 (Table 5). Supplemental Table S2 provides detailed information on the aggregated Fst for each type of tree during GS 2023. In the first scenario, POD0 varied between 8.2 and 15.0 mmol m^−2^, with lower values typical for pines in the FH zone. The POD0 for pines in the ATE zone was slightly greater, whereas the highest POD0 was modelled for *P. abies* and *A. alba* in the FH zone (Table 5).

**Table 5:** Model estimation of POD0 uptake by the analysed Pinaceae species at ambient O_3_ concentrations in the 2023 growing season and the projected critical levels considering an OxS threshold of -0.05 (CL(OxS))

Zone	Pinaceae species	Projected for ambient O_3_ concentrations		Projected for CL(OxS)	
		avgO_3_ (ppb)	POD0 (mmol m^−2^)	avgO_3_ (ppb)	POD0 (mmol m^−2^)
ATE	*Pinus mugo*	52.7	10.9	158.5	32.8
	*Pinus cembra*	:	12.8	174.6	42.5
FH	*Pinus mugo*	27.5	8.2	116.0	32.2
	*Pinus cembra*	:	9.9	141.1	47.4
	*Pinus sylvestris*	:	9.9	137.8	39.4
	*Picea abies*	:	15.0	124.1	62.4
	*Abies alba*	:	15.0	165.3	83.2

In the second scenario, where O_3_ concentrations were projected to cause a 5% decrease in OxS, POD0 ranged from 32 to 84 mmol m^−2^, primarily because of the difference in O_3_ concentration. The seasonal avgO_3_ derived from an ozonation experiment for a 5% decrease in OxS was several times greater than the seasonal avgO_3_ under natural conditions (Table 5). The POD0 projected for O_3_ concentrations that could cause a 5% decrease in OxS was considered a critical-level CL(OxS).

**Fig. 6:**
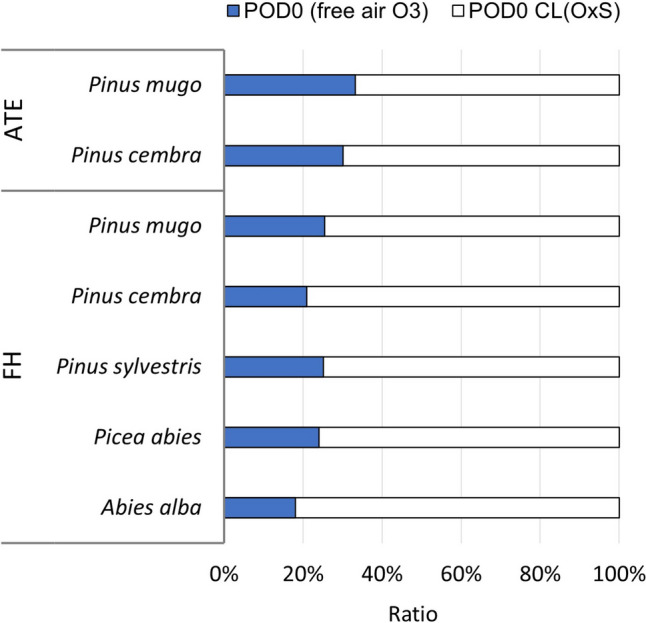
O_3_ tolerance potential utilisation of the Pinaceae species, expressed as a percentage of POD0 uptake under ambient O_3_ conditions during the 2023 growing season (POD0-free air O_3_) compared with the projected critical level considering an OxS threshold of -0.05 (POD0 CL(OxS))

The significant contrast between POD0 for ambient O_3_ conditions and CL(OxS) highlights the low sensitivity of adult individuals of Pinaceae species to ozone in the natural environment of the High Tatra region. The ratio between POD0 uptake by trees under ambient O_3_ conditions and CL(OxS) (Fig. 6) served as a marker of the utilisation of O_3_ tolerance potential. Ozone tolerance potential refers to the ability of a plant or tree to withstand and mitigate the harmful effects of O_3_ exposure without sustaining significant damage. Starting with the lowest values of CL(OxS) projected for *P. mugo*, which reached 32.2 mmol m^−2^ and 32.8 mmol m^−2^ for FH and ATE, respectively, the data indicated differences in O_3_ tolerance potential between zones at different altitudes. The O_3_ tolerance potential of *P. mugo* was utilised to 25.5% for the FH zone and 33.2% for the ATE zone (Fig. 6). *P. sylvestris* FH followed *P. mugo*, with a CL(OxS) of 39.4 mmol m^−2^ and an O_3_ tolerance potential utilisation of 25.1%. For *P. cembra*, the CL(OxS) values were 42.5 mmol m^−2^ and 47.4 mmol m^−2^ for the ATE and FH zones, respectively, suggesting greater resistance to O_3_ injury in the FH zone than in the ATE zone. This was confirmed by the O_3_ tolerance potential utilisation values of 20.1% in the FH zone and 30.1% in the ATE zone. A high CL(OxS) was projected for *P. abies* FH, reaching 62.4 mmol m^−2^. With an ambient POD0 of 15.0 mmol m^−2^, the O_3_ tolerance potential of this species was utilised to 24.0%. The highest CL(OxS) among the analysed species was found in *A. alba* FH, with 83.2 mmol m^−2^. Given the ambient POD0 of 15.0 mmol m^−2^, *A. alba* FH utilised only 18.0% of its O_3_ tolerance potential. Generally, the ratio of less than 20% for *A. alba* and 20–40% for the other Pinaceae species (Fig. 6) suggested that these trees could tolerate POD0 levels approximately 60–80% higher than those currently measured in their natural environment without a significant risk of damage.

## Discussion

### Injury index—response to stress agents

Plants in mountain environments absorb surface ozone (O_3_), a secondary air pollutant formed in the lower atmosphere through chemical reactions from both anthropogenic and biogenic sources (Li et al. 2024). Evergreen mountain conifers, which retain their foliage for years, take up O_3_ all year. However, the adverse effects of O_3_ are more pronounced during the active growing season, when the stomatal conductance and assimilation processes peak. The climatological conditions at elevated positions in the High Tatra Mts allow the onset of vegetation phenological phases after snowmelt, typically occurring in the second half of May, and the GS lasts until the temperature drops below 5 °C, which usually occurs in October (Lukasová et al. 2023). According to meteorological measurements, GS 2023 started approximately one month earlier at FH (in May) than at ATE (in June) and lasted until the end of September. The GS length corresponds to the “green window”—the period when no days with all-day hourly air temperatures below 5 °C were recorded (Fig. S1). Despite the shorter length of the GS at ATE, seasonal ambient O_3_ exposure, represented by sumO_3_ and avgO_3_, reached higher levels than those in the FH zone (Table 5).

The INX(1) values summarised in Table 2, which are related to injury from the ambient environment, suggested that needle samples of *P. mugo* and *P. cembra* collected at ATE had indices slightly higher than those from FH but substantially lower than those of *P. abies* FH. The higher INX(1) in *P. abies* needles than in other species can be attributed primarily to their physiological and anatomical characteristics, since the trees were growing under suitable microclimatic and environmental conditions, and only healthy-looking individuals were sampled. INX(2–6) exhibited a clear injury increase due to the selective effect of artificial O_3_ during the ozonation process for all the Pinaceae species examined (Table 2).

For the calculation of OxS after ozonation, it was necessary to derive the injury index of the control samples. Performing measurements on control samples from mature trees growing under similar environmental conditions but without O_3_ exposure in ambient air would be difficult, especially given that this study was performed in the protected area of Tatra National Park. Given these circumstances, the injury index values for the control samples, marked as INX(0), were modelled. To define the INX(0) for the injury corresponding to sumO_3_ = 0 ppm, the scatter plot showing the relationships between INX(*n*) and increasing sumO_3_ was fitted by an exponential function (Fig. 2).

### Changes in chemical composition induced by O_3_

The chemical composition of terpenoids in conifers is dynamic and can change depending on the type of environmental stress to which the tree is exposed (Zulak and Bohlmann 2009). The biosynthesis of terpenoids in conifer defence involves the formation of isoprenoid (C_5_) units and their elongation to prenyl diphosphates (C_10_, C_15_, and C_20_), which serve as substrates for terpene synthases (Keeling and Bohlmann 2006). Isoprene plays a role in enhancing tolerance to ozone and other reactive oxygen species (Sharkey et al. 2007; Wang et al. 2016) by preventing visible damage caused by O_3_ exposure (Loreto and Velikova 2001; Loreto et al. 2001) and mitigating the loss in photosynthetic capacity due to ROS (Peñuelas and Llusià 2002; Peñuelas et al. 2005). GC–MS analyses revealed that sesquiterpenes (C_15_H_24_) had the highest percentages in the chemical composition of the samples, and these levels remained high even after ozonation (Fig. 3). This suggested the robustness of C_15_H_24_ against intense oxidation. In contrast, ozonation contributed to the breakdown of monoterpenes (C_10_H_16_), whose percentages significantly decreased with increasing exposure to artificial O_3_. However, the expected oxidation products, such as alcohols, aldehydes, ketones or MDA (Davey et al. 2005; Pellegrini et al. 2021), have not been confirmed. Similar findings were reported by Hůnová et al. (2010), who hypothesised that higher ambient O_3_ exposure would result in increased MDA contents in *P. abies* needles under real conditions, which was not confirmed. In our study, the TLE of *P. abies* processed via the same method as the other species did not yield interpretable results. Future analyses could benefit from further chemical fractionation via saponification using 6% KOH in methanol, resulting in acidic and neutral fraction differentiation (Fornace et al. 2014; Freimuth et al. 2017; Bechtel et al. 2018; Žatková et al. 2023). In samples from other species, instead of the expected products, we observed a significant increase in the proportion of C_7–8_ hydrocarbon oxidation products, particularly benzoic acid, in the artificially ozonised samples (Fig. 4).

**Fig. 7:**
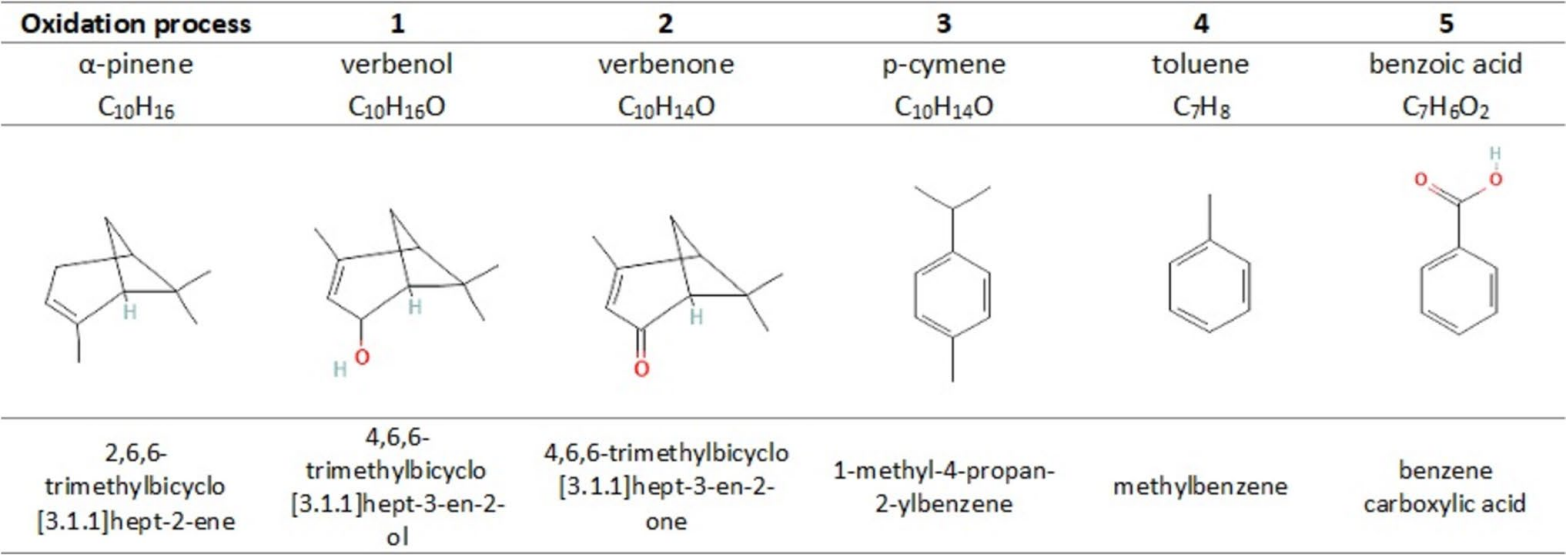
Oxidative cleavage products in the multistep oxidation process of α-pinene leading to the formation of benzoic acid. The chemical names of the compounds are based on the International Union of Pure and Applied Chemistry (IUPAC) nomenclature standards

The reaction scheme in Fig. 7 illustrates how multistep oxidative pathways can transform α-pinene, a widely occurring monoterpene found in pine resin, to benzoic acid as one of the final products. The oxidation process includes five steps with the following oxidative products: (1) verbenol, (2) verbenone, (3) *p*-cymene, (4) toluene, and (5) benzoic acid. Generally, O_3_ reacts with the double bond of the monoterpene through ozonolysis reactions, known as the Criegee mechanism (Almatarneh et al. 2018), leading to the cleavage of double bonds and the formation of primary ozonides, smaller aldehydes and ketones.

Primary oxidative products such as verbenol and verbenone are obtained after α-pinene undergoes oxidation with mild oxidising agents (Grzeszczak et al. 2023; Petrović et al. 2022). The formation of secondary ozonides, including *p*-cymene, toluene, and benzoic acid, may require stronger oxidative conditions, such as the presence of catalysts, specific oxidising agents, and controlled reaction environments. Golets et al. (2013) demonstrated the feasibility of producing ρ-cymene from abundant α-pinene via bimetallic heterogeneous catalysts. Interestingly, Gratien et al. (2011) reported the unexpected formation of *p*-cymene during the oxidation of α-pinene in air by atmospheric oxidants (hydroxyl radicals, ozone, and nitrate).

The conversion of *p*-cymene to toluene involves the removal of the isopropyl group attached to the benzene ring. This process can be achieved through various catalysed reactions, such as contact of *p*-cymene with benzene in the presence of a trans-alkylation catalyst (Thai et al. 2013) or the use of a catalyst for flow disproportionation reactions that produce a mixture of toluene and propylene from cymene (Tadepalli et al. 2022). Toluene, also known as methylbenzene, is an aromatic hydrocarbon characterised by a methyl group attached to a benzene ring. This structural feature significantly influences its chemical behaviour, laying the groundwork for its conversion to benzoic acid through catalytic oxidation of the methyl group, leading to the formation of benzaldehyde and further benzoic acid (Zhu et al. 2024).

### Oxidative stability determination

Oxidative stability refers to the resistance of chemical substances to oxidation by air. The OxS index is commonly used to quantify the relative resistance of fats and oils to oxidation, as the double bonds of unsaturated fatty acid methyl esters are particularly susceptible to this process (Kumar and Sharma 2015). In conifer trees, oxidative resistance is bolstered by terpenes with antioxidant properties, which protect the plant from pathogens and herbivores through constitutive or induced defence mechanisms. Ozonolysis, the reaction between ozone and alkene substances in conifer needles, plays a significant role in the degradation and alteration of the structures within the assimilation apparatus. As a powerful oxidant, O_3_ can cleave alkenes and insert oxygen into a C–H bond to form an oxidative product (Hanson 2017). We assumed that ozonolysis of needle terpenes, as induced by ozonation in our manipulative experiment, could be quantified via the OxS indicator, which, to our knowledge, has not yet been applied to determine the effects of O_3_ on conifer trees. The OxS values of needles vary significantly among tree species, which may be attributed to several factors—the anatomical and physiological differences in needle structure between the analysed species, e.g., cuticle thickness, stomatal conductance, antioxidant levels, and nutrient status (Fares et al. 2010; Li et al. 2016; Manzini et al. 2024). Specific growing conditions and climates also influence the extent of O_3_ injury (Ferretti et al. 2024). Consequently, some species may have lower thresholds for ozone damage, making them more susceptible even at lower ozone concentrations. In our study, *P. mugo* was the most susceptible species to O_3_ injury, exhibiting the lowest OxS at both the FH and ATE sites across the ozonation series. In contrast, *A. alba* presented the highest OxS (Table 4). Ferretti et al. (2018) suggested that forests exposed to high ozone levels and other oxidative stresses for decades may have developed an acclimation mechanism to protect themselves.

### Proposing critical levels

Critical levels for vegetation are typically expressed as thresholds for concentration, cumulative exposure, or cumulative stomatal flux of atmospheric pollutants above which direct adverse effects on sensitive vegetation are likely to occur, based on current knowledge (CLRTAP 2017). Stomatal O_3_ flux accumulated over the growing season (POD_Y_) is now considered the most biologically relevant metric for assessing O_3_ injury to vegetation and establishing CLs (Paoletti et al. 2022). To develop CLs, studies have examined the potential effects of O_3_ on tree biomass loss and carbon sequestration via O_3_ free-air controlled exposure (FACE) experiments (e.g., Pregitzer and Talhelm 2013; Zhang et al. 2018; Hoshika et al. 2024; Manzini et al. 2025). However, these experiments may not fully represent actual field conditions (Juráň et al. 2021). In contrast, the occurrence of visible foliar injury in the field can serve as a reliable proxy for biomass loss, especially when epidemiology-based CLs are set (Paoletti et al. 2022). This approach is particularly relevant in southern European forests, where ground-level O_3_ poses a significant health risk to ecosystems (Sicard et al. 2016). The risk of O_3_ damage is not uniform across all biogeographic regions in Europe. Observations of visible O_3_ injury in mountain conifers in Eastern Central Europe suggested that ambient O_3_ concentrations in this region may have a lower impact on native vegetation than previously assumed (Matoušková et al. 2010; Bičárová et al. 2023). Plant stomatal function varies by species and region, reflecting the different adaptations of plants to climate and soil conditions. Therefore, biogeographical regions should be considered when deriving CLs. Currently, no CLs are available for alpine regions influenced by the continental climate (CLRTAP 2017).

This study proposed O_3_ flux-based critical levels against OxS (CL(OxS)) to track cell membrane injury in needle samples collected under field conditions. The laboratory manipulative experiment revealed a selective response of the plant material to O_3_ exposure, isolating it from the influence of other stressors. The analysis revealed a decrease in OxS(n) in response to gradually increasing artificial sumO_3_ exposure (Tables 2 and 4). Regression analysis between OxS (*n*) and sumO_3_ (Fig. 5) allowed us to derive the seasonal sumO_3_, which, when recalculated considering the length of the growing season, represents the seasonal average hourly O_3_ concentration useful for POD0 modelling. For subalpine and alpine zones, where a nearly flat daily O_3_ pattern is observed (Bičárová et al. 2020), the avgO_3_ concentration for the growing season can be used in place of the hourly O_3_ concentration in the model input file. Monitoring in the High Tatras revealed that the daily amplitude of O_3_ gradually decreases with increasing altitude, leading to a fairly steady daily course of O_3_ at relatively high altitudes (Bičárová et al. 2005). Similar findings were reported by Štefánik and Šedivá (2022) for two high-altitude regional background stations (Chopok, 1958 m asl, and Kojšovská hoľa, 1230 m asl), where nearly constant O_3_ concentrations were observed throughout the day, with a slight decrease in the morning hours. Using the constant seasonal avgO_3_ in POD0 modelling allowed us to project CL(OxS) for the defined threshold value of OxS = -0.05 (Table 5), which represents up to 5% injury to cell membrane integrity induced by selective O_3_ effects. The constant seasonal avgO_3_ derived for this threshold was greater than the constant 10 ppb O_3_ estimated as the preindustrial mean O_3_ concentration, which should be used as a reference value for determining O_3_ flux-based critical levels (CLRTAP 2017).

The effect of O_3_ on vegetation can be evaluated in several ways. In general, for conifers, attention is often focused on *P. abies*, one of the key species in Europe both economically and ecologically. Its detoxification capacity was expressed by the threshold *Y* = 1 mmol m^−2^, and modelled values of POD_1_ can be assessed according to CLs of 8 mmol m^−2^ and 9.2 mmol m^−2^ (CLRTAP 2017), which correspond to an acceptable biomass loss, i.e., a 2% reduction in annual new growth. The EMEP report (2020) indicated that in 2018, a CL of 8 mmol m^−2^ for POD_1_ was exceeded across most of Europe, although these modelled levels could not be fully validated by observations. Sicard et al. (2016) emphasised the need to validate CLs for POD_Y_ and develop new flux-based critical levels of CLef for forest protection against visible O_3_ injury. To avoid underestimating real O_3_ uptake, the use of POD0 was recommended to define CLef for O_3_-sensitive Pinaceae conifers. Based on visible foliar injury, *P. cembra* was identified as highly O_3_ sensitive, with CLef values of 19 mmol m^−2^, whereas moderately O_3_ sensitive *Pinus halepensis* had a CLef of 32 mmol m^−2^. In our study, the CL(OxS) values for *P. cembra*, 42.5 mmol m^−2^ at ATE and 47.4 mmol m^−2^ at FH were higher than the published CLef values, suggesting greater vulnerability for *P. cembra* in the Mediterranean biogeographical region. CL(OxS) values slightly above 30 mmol m^−2^ and just below 40 mmol m^−2^ for *P. mugo* and *P. sylvestris*, respectively, indicated lower tolerance to O_3_ than did *P. cembra*. The highest CL(OxS) values were observed for the more tolerant species *A. alba* and *P. abies*, although *P. abies* presented very high initial injury rates. In general, based on O_3_ tolerance potential utilisation, which refers to the ability of a plant or tree to withstand and mitigate the harmful effects of O_3_ exposure without sustaining significant damage, the Pinaceae species included in this study could tolerate POD0 levels approximately 60–80% higher than those currently measured in the natural environment. These high tolerance values of Pinaceae species indicate their ability to resist oxidative stress, which is essential for maintaining health, productivity, and ecosystem services in ozone-impacted areas.

Several studies have analysed the sensitivity of Pinaceae family trees to O_3_ (Sicard et al. 2016; Lukasová et al. 2022; Bičárová et al. 2023). Wieser et al. (2006) summarised the impacts of O_3_ on adult *P. cembra* in the timberline ecotone for different regions of Europe and reported that exposure to ambient and twice-ambient O_3_ throughout one growing season caused visible injury or affected the photosynthetic machinery and biochemical parameters in current to 1-year-old needles in the central European Alps. Huttunen and Manninen (2013) reported that *P. sylvestris* might be considered an O_3_-sensitive conifer species, with mature pines being more sensitive than younger trees. A slight trend towards greater O_3_ tolerance among evergreen or coniferous species could be attributed to anatomical characteristics or foliage type-specific differences in specific leaf areas (Bergmann et al. 2017). The forest response to O_3_ is a complex interplay of individual tree responses across different species and sizes. Additionally, O_3_ effects on plant‒plant, plant–insect, and plant‒microbe interactions can alter plant competitiveness due to varying degrees of susceptibility among plants (Agathokleous et al. 2022).

## Conclusions

This study presented the results of a manipulative laboratory experiment that proposed O_3_ flux-based critical levels for Pinaceae species via a new oxidative stability indicator. The injury index (INX), determined via the modified EL method, was used to quantify changes in cell membrane integrity due to oxidative stress. Chemical analyses of needle samples revealed significant changes: a decrease in monoterpenes (C_10_H_16_) and an increase in oxidative forms (C_7‒8_H_n_O_n_), particularly benzoic acid (C_7_H_6_O_2_), although the presence of expected products such as malondialdehyde was not confirmed.

Injury levels were assessed in two contexts: (i) the effect of multiple stress factors on needles in the outdoor environment during the growing season (INX(1)) and (ii) injury from artificial ozonation in controlled indoor conditions without additional environmental stress agents (INX(2–6)). The initial injury index without O_3_ exposure (INX(0)) was modelled. These injury levels are crucial for determining OxS and the stomatal O_3_ flux-based critical level (CL(OxS)). The threshold value of OxS (-0.05), corresponding to a 5% injury of needle samples due to O_3_, was defined as the level at which O_3_ can start causing serious disturbances in forest trees.

The derived O_3_ exposure doses, which were converted to seasonal O_3_ concentrations for the OxS -0.05 threshold, were used in the modelling of the stomatal O_3_ flux dose (POD0), which represented CL(OxS). The significantly lower POD0 modelled for real ambient O_3_ conditions during the 2023 growing season compared with CL(OxS) indicated that Pinaceae species could be vulnerable to substantially higher ground-level O_3_ than the ambient concentrations recorded by long-term monitoring in the High Tatras. The ratio between POD0, which represents stomatal O_3_ uptake under real conditions, and CL(OxS) demonstrated the low sensitivity of adult individuals of Pinaceae species growing in the High Tatras, a part of the Alpine biogeographical region in Eastern Central Europe, to current ozone pollution.

## Supplementary Information

Below is the link to the electronic supplementary material.

Supplementary file1 (DOCX 615 KB)

## Data Availability

Data are available upon request from the authors. The data that support the findings of this study are available from the corresponding author, Veronika Lukasová, upon reasonable request.

## Abbreviations

*sumO*_3_: Summed O_3_ concentration
*avgO*_3_: Averaged O_3_ concentration
*CL*: Critical level
*CL(OxS)*: Critical level based on oxidative stability indicator
*DO*_3_*SE*: Dry deposition model designed to estimate the stomatal deposition (or stomatal flux, Fst) of O_3_ to selected European land-cover types and plant species
*EL*: Electrolyte leakage
*GC-MS*: Gas chromatography-mass spectrometry
*TLE*: Total lipid extract
*POD0*: Phytotoxic ozone dose without detoxification threshold limit
*OxS*: Oxidative stability
*INX*: Injury index
*INX(0)*: Injury index without O_3_ pollution
*INX(1)*: Injury index related to the effect of surface O_3_ in the natural environment during the growing season
*INX(2–6)*: Injury index related to the effect of controlled artificial O_3_ exposure under additional graduated ozonation in the laboratory setting
