# Supplementary material for: Indication of the sensitivity of Pinaceae species growing in Eastern Central Europe to ground-level ozone pollution
Source: Environ Sci Pollut Res Int. 2025 Jan 14;32(5):2638–55. doi: 10.1007/s11356-025-35905-7 (PMC11802678; doi:10.1007/s11356-025-35905-7)
Supplement: Supplementary file 1 — Supplementary file1 (DOCX 615 KB) [file 11356_2025_35905_MOESM1_ESM.docx]

**Supplementary Information – Environmental Science and Pollution Research**

**Indication of the Sensitivity of *Pinaceae* Species Growing in Eastern Central Europe to Ground-level Ozone Pollution**

Veronika Lukasová^1,*^, Svetlana Varšová^1^, Lucia Žatková^2^, Katarína Adamčíková^3^, Anna Buchholcerová^1^, Milan Onderka^1,4^, Rastislav Milovský^2^, Dušan Bilčík^1^, Veronika Mináriková^4^

^1^ Earth Science Institute, Slovak Academy of Sciences, Dúbravská cesta 9, Bratislava 840 05, Slovakia

^2^ Earth Science Institute, Slovak Academy of Sciences, Ďumbierska 1, Banská Bystrica 974 11, Slovakia

^3^ Institute of Forest Ecology, Department of Plant Pathology, and Mycology, Slovak Academy of Sciences, Akademická 2, Nitra 949 01, Slovakia

^4^ Slovak Hydrometeorological Institute, Jeséniova 17, Bratislava 833 15, Slovakia

* Corresponding author: Veronika Lukasová, email: [geofluka@savba.sk](mailto:geofluka@savba.sk), tel.number: +421-52-7879146

**Table S1** DO_3_SE model parameterisation scheme according to the built-in pre-set for Boreal-Coniferous forests (Norway spruce) with modification of *selected parameters (Bičárová et al. 2019; Buchholcerová et al. 2021). Parametrisation for *P. sylvestris* such a*s P. cembra* and *A. alba* such as *P. abies* was employed

| **Vegetation characteristics** | Norway spruce  *Picea abies* | Mountain pine  *P. mugo* | Swiss pine  *P. cembra* |
| --- | --- | --- | --- |
| H (m): Canopy height | 20 | 2 | 20 |
| Root (m): Root depth | 1.0 | 1.0 | 1.0 |
| Lm (m): Cross-wind leaf dimension | 0.01 | 0.01 | 0.01 |
| Albedo (fraction) | 0.12 | 0.12 | 0.12 |
| G_max_ (mmol O_3_ m^-2^ PLA s^-1^) | 125 | 110* | 113* |
| Sun/shade factor (fraction) | 1.0 | 1.0 | 1.0 |
| f_min_ (fraction): Minimum stomatal conductance to O_3_ | 0.1 | 0.1 | 0.1 |
| R_ext_ (s m^-1^): External plant cuticule resistance | 2500 | 2500 | 2500 |
| Threshold Y for PODy (nmol m^-2^ s^-1^) | 0 | 0 | 0 |
| G_sto0_ (µmol m^-2^ s^-1^): Closed stomata conductance | 30,000 | 30,000 | 30,000 |
| m (dimensionless): Species-specific sensitivity to An | 16.83 | 16.83 | 16.83 |
| Vcmax (µmol m^-2^ s^-1^): Maximum catalytic rate at 25°C | 30.00 | 30.00 | 30.00 |
| Jcmax (µmol m^-2^ s^-1^): Maximum rate of electron transport at 25°C | 60.00 | 60.00 | 60.00 |
| LAI_a (m^2^ m^2^): Leaf area index | 6.5 | 6.5 | 6.5 |
| SAI (surface area index) calculation | Forest | Forest | Forest |
| **Environmental response** |  |  |  |
| light_a_ (dimensionless): Species-specific parameter for response G_sto_ to photosynthetic photon flux density (PPFD) | 0.006 | 0.006 | 0.0032* |
| T_min_ (°C): Minimum temperature for G_sto_ | 0 | 0 | 0 |
| T_opt_ (°C): Optimum temperature for G_sto_ | 20 | 20 | 27* |
| T_max_(°C): Maximum temperature for G_sto_ | 100 | 100 | 100 |
| VPD_min_ (kPa): Vapour pressure deficit for min. G_sto_ | 2.80 | 2.80 | 2.50* |
| VPD_max_ (kPa): Vapour pressure deficit for max. G_sto_ | 0.80 | 0.80 | 0.25* |
| SWP_min_ (MPa): Soil water potential for min. G_sto_ | -1.5 | -1.5 | -1.5 |
| SWP_max_ (MPa): Soil water potential for max. G_sto_ | -0.7 | -0.7 | -0.7 |
| **Model options** |  | | |
| Stomatal conductance model | Multiplicative | | |
| fO_3_calculation | Not used (fO_3_=1) | | |
| Soil water influence on G_sto_ | Use fSWP | | |
| fSWP calculation | Linear (SWP_min, SWP_max) | | |
| **Season** | Foothill (FH) 01/05/ – 30/09/2023  Alpine treeline ecotone (ATE) 01/06/ – 30/09/2023 | | |

**Table S2** Statistics of hourly O_3_ and meteorological input data over the growing season (GS) for foothill (FH) and alpine treeline ecotone (ATE) zones

| Input variables | | FH | ATE |
| --- | --- | --- | --- |
| O_3_ concentration (ppb) | Mean | 27.5 | 52.7 |
|  | Max | 65.5 | 76.2 |
|  | Min | 4.0 | 25.8 |
|  | STD | 9.0 | 7.1 |
|  | Sum (ppm) | 101.0 | 154.4 |
| Air temperature  T (°C) | Mean | 15.3 | 11.5 |
|  | Max | 29.0 | 22.0 |
|  | Min | 2.2 | 0.0 |
|  | STD | 9.0 | 3.9 |
| Vapour pressure deficit  VPD (kPa) | Mean | 0.3 | 0.2 |
|  | Max | 2.1 | 1.3 |
|  | Min | 0.0 | 0.0 |
|  | STD | 0.4 | 0.2 |
| Precipitation amount P (mm) | Sum | 545.8 | 712.7 |
| Global radiation R (kW m^–2^) | Sum | 622.8 | 494.7 |

**
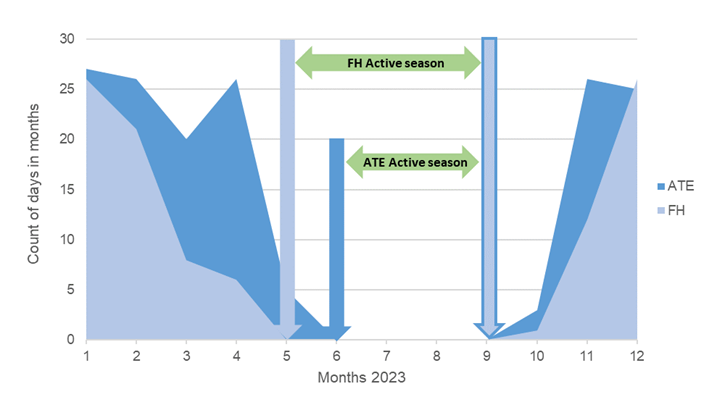
**

**Fig. S1** The GS season according to the meteorological thermal threshold air temperature of 5°C for foothill (FH) and alpine treeline ecotone (ATE) zones. The GS length corresponds to the green window of months with the absence of days with all-day hourly air temperature < 5°C


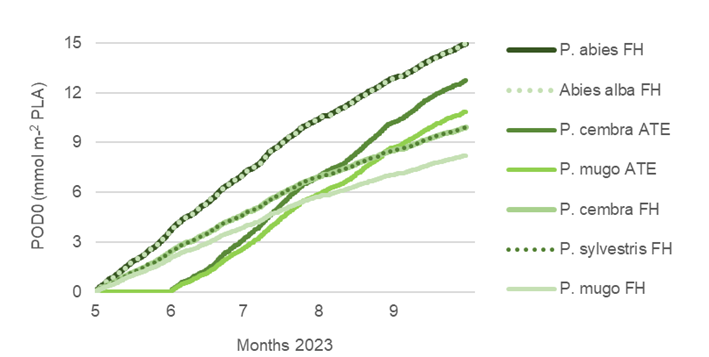


**Fig. S2** Seasonal course of stomatal O_3_ flux (Fst) aggregated over GS 2023 to the value of POD0 for foothill (FH) and alpine treeline ecotone (ATE) forest trees

**NOTES: Modelling of stomatal O_3_ flux using the DO_3_SE model**

DO_3_SE is a dry deposition model designed to estimate stomatal flux Fst of O_3_ to selected European land-cover types and plant species (SEI 2023). The Fst accumulated during active plant growth is defined as PODY, (nmol m^−2^ PLA s^−1^, PLA is the projected leaf area) i.e., the Phytotoxic Ozone Dose above a threshold flux of Y determined separately for different types of woody plants. Due to the absence of research experiments to define the threshold flux reflecting local specific conditions in the High Tatra Mts. region, we assumed Y = 0. The core of the DO_3_SE model is a multiplicative algorithm that considers the influence of four basic environmental variables: air temperature AT (°C), vapour pressure deficit VPD (kPa), light or irradiance R (W m^-2^), and soil water potential SWP (MPa) on the maximal stomatal conductance G_max_ (mmol O_3_ m^-2^ PLA s^-1^) in the frame of model parameterisation scheme (**Tab. S1**., CLRTAP 2017). DO_3_SE model processed hourly O_3_ and meteorological input data (**Tab. S2**) monitored by automatic UV Absorption O_3_ analysers and weather stations (AWS) Physicus during the growing season (GS) period. GS lasting from the 1^st^ of May to the 30^th^ of September 2023 at the foothill (FH) location and from the 1^st^ of June to the 30^th^ of September 2023 at the elevated alpine treeline ecotone (ATE) site (**Fig. S1**). The start of GS corresponded to the start of the meteorological season for the active growth of plants (Korner et al. 2023) and the end of GS agreed to the time of sample collections. The course of model results of Fst (nmol m^-2^ s^-1^) aggregated over GS for tree species types in FH and ATE zones is shown in **Fig. S2**.

**References:**

Bičárová S, Sitková Z, Pavlendová H, Fleischer P, Fleischer Jr P, Bytnerowicz A (2019) The role of environmental factors in ozone uptake of *Pinus mugo* Turra. Atmos Pollut Res. <https://doi.org/10.1016/j.apr.2018.08.003>

Buchholcerová A, Fleischer Jr P, Štefánik D, Bičárová S, Lukasová V (2021) Specification of Modified Jarvis Model Parameterization for *Pinus cembra*. Atmos. <https://dx.doi.org/10.3390/atmos12111388>

CLRTAP (2017) Mapping Critical Levels for Vegetation, Chapter III of Manual on methodologies and criteria for modelling and mapping critical loads and levels and air pollution effects, risks and trends. UNECE Convention on Long range Transboundary Air Pollution. <https://unece.org/fileadmin/DAM/env/documents/2017/AIR/EMEP/Final__new_Chapter_3_v2__August_2017_.pdf>. Accessed 14 August 2024

Körner C, Möhl P, Hiltbrunner E (2023) Four ways to define the growing season. Ecol Let. <https://doi.org/10.1111/ele.14260>

SEI (2023) DO3SE (Deposition of ozone for stomatal exchange). <https://www.sei-international.org/do3se>. Accessed 10 July 2024

**Table S3.** List of chemical substances identified by CG-MS method in Pinaceae species needle samples collected in field conditions in the High Tatra Mts. region. *NoCAS is a unique identification number of a chemical substance to index the substance in the Chemical Abstracts Service (CAS) registry.

| No. | RI | Formula | Name | Structure | *NoCAS |
| --- | --- | --- | --- | --- | --- |
|  |  | C_10_ | monoterpenes |  |  |
| I | 1011 | C_10_H_16_ | γ-terpinene | 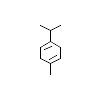 | 99-85-4 |
| II | 1042 | C_10_H_16_ | α-thujene | 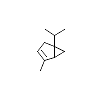 | 2867-05-2 |
| III | 1079 | C_10_H_16_ | α-pinene | 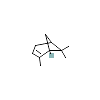 | 7785-70-8 |
|  |  | C_7-8_ H_n_O_x_ | oxidation products |  |  |
| IV | 1213 | C_7_H_6_O_2_ | benzoic acid | 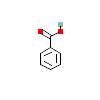 | 65-85-0 |
| V | 1246 | C_8_H_8_O | coumaran | 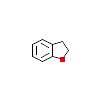 | 496-16-2 |
|  |  | C_15_ | sesquiterpenes |  |  |
| VI | 1354 | C_15_H_24_ | δ-elemene | 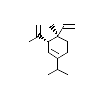 | 20307-84-0 |
| VII | 1442 | C_15_H_24_ | α-cubebene | 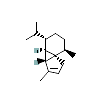 | 17699-14-8 |
| VIII | 1506 | C_15_H_24_ | germacrene D | 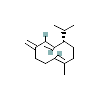 | 23986-74-5 |
| IX | 1537 | C_15_H_24_ | γ-cadinene | 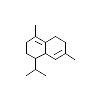 | 39029-41-9 |
| X | 1544 | C_15_H_24_ | δ-cadinene | 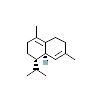 | 483-76-1 |
|  |  | C_20+_H_n_O_x_ | oxidation products |  |  |
| XI | 2381 | C_21_H_30_O_3_ | dehydroabietic acid methyl ester | 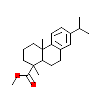 | 1235-74-1 |
| XII | 2845 | C_21_H_30_O_3_ | dehydroisoandrosterone acetate | 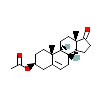 | 853-23-6 |
| XIII | 3271 | C_29_H_50_O_2_ | α-tocopherol | 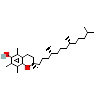 | 10191-41-0 |


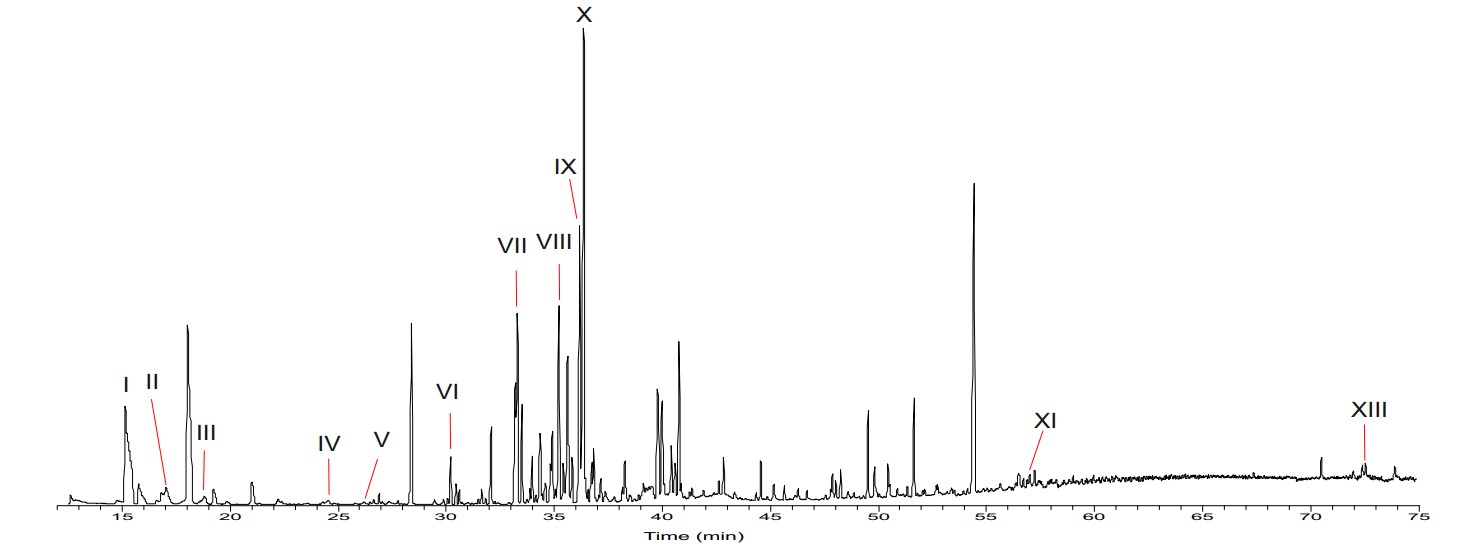


**Fig. S3 (a)** Chromatogram graph for sample A (above) and changes in chemical composition between samples A, B and C (bellow) in *Pinus mugo* ATE


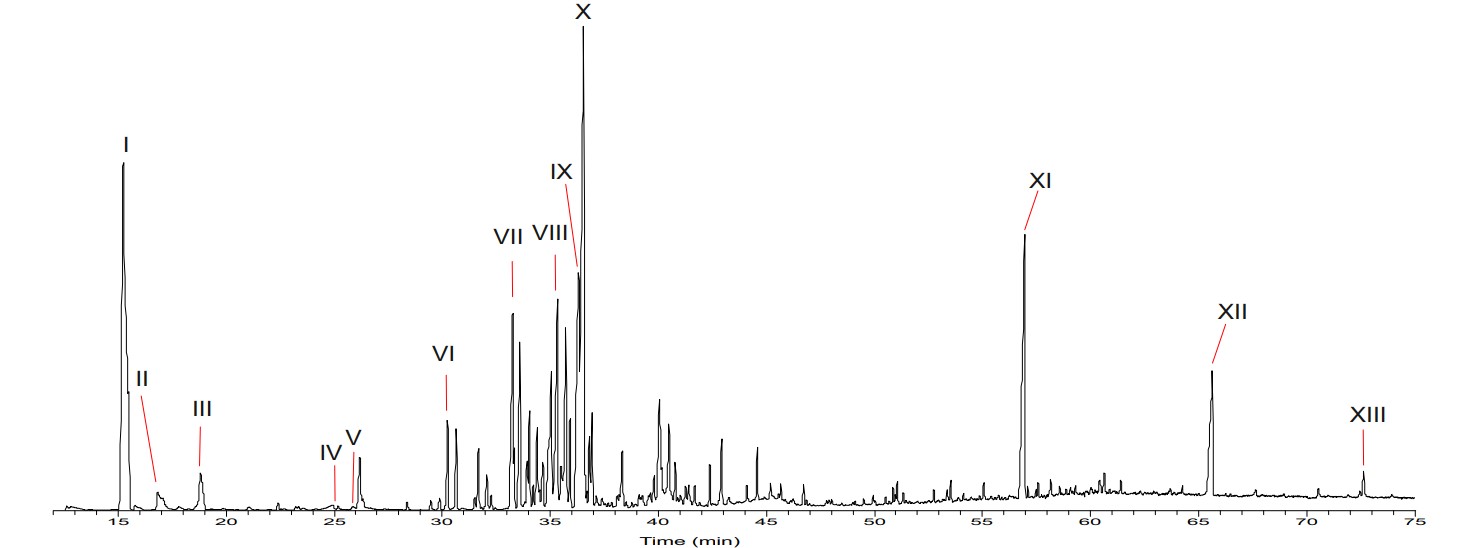


**Fig. S3 (b)** Chromatogram graph for sample A (above) and changes in chemical composition between samples A, B and C (bellow) in *Pinus cembra* ATE


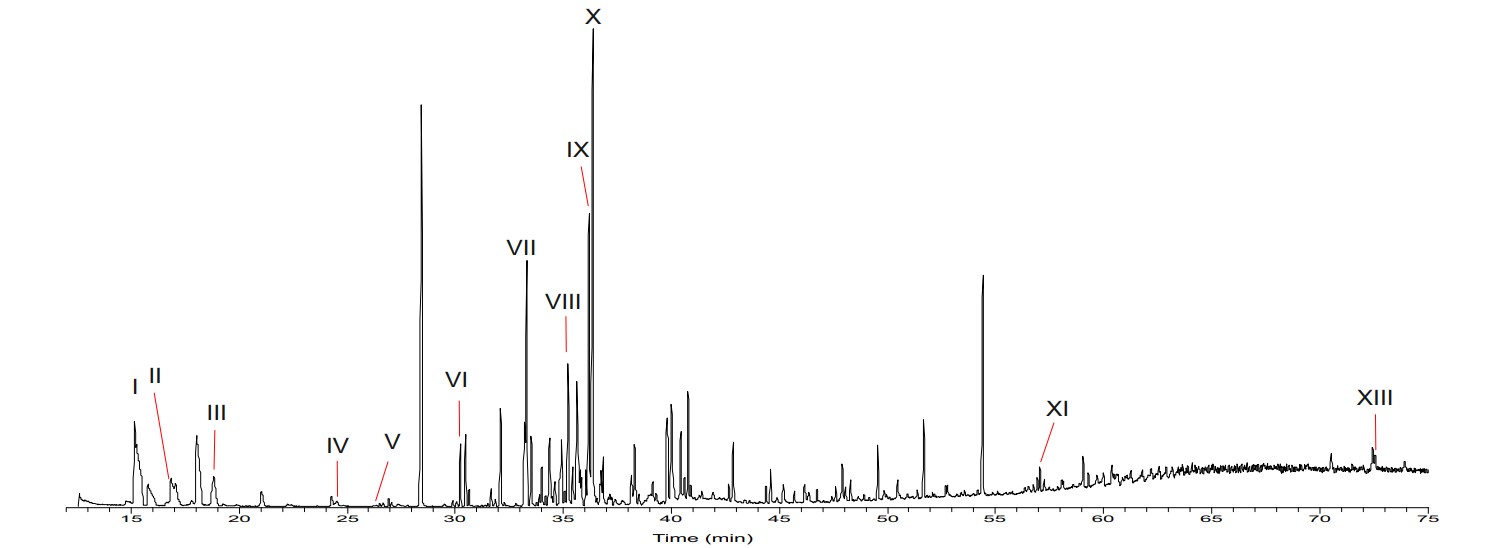


**Fig. S3 (c)** Chromatogram graph for sample A (above) and changes in chemical composition between samples A, B and C (bellow) in *Pinus mugo* FH


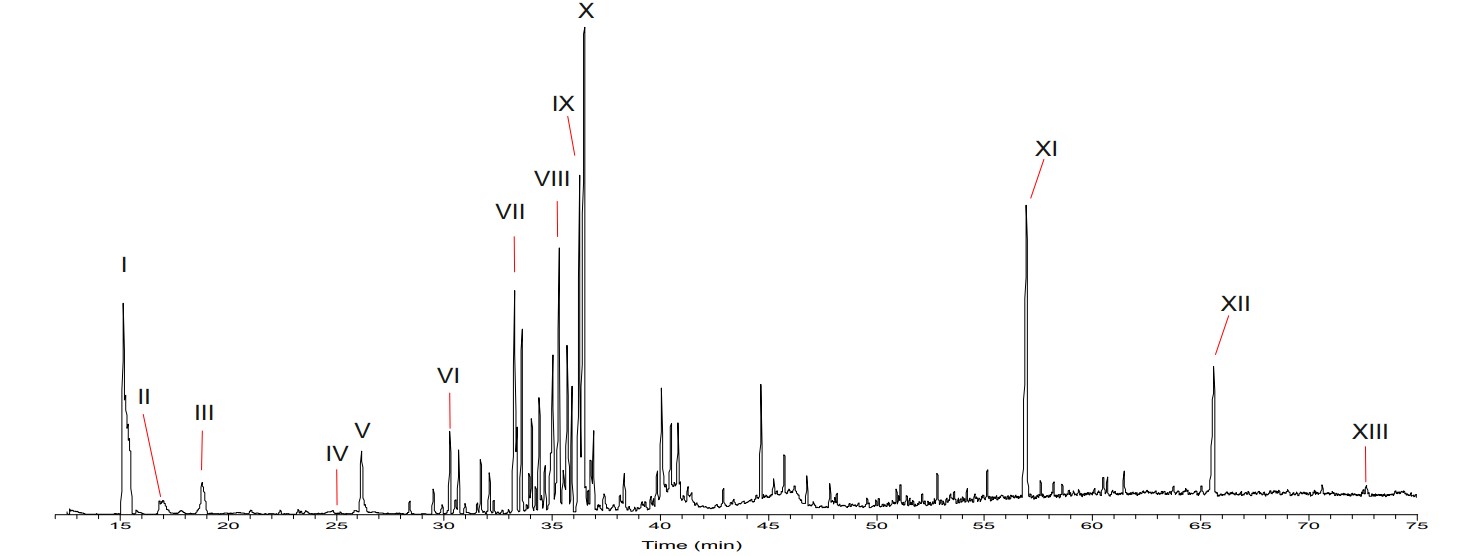


**Fig. S3 (d)** Chromatogram graph for sample A (above) and changes in chemical composition between samples A, B and C (bellow) in Pinus cembra FH


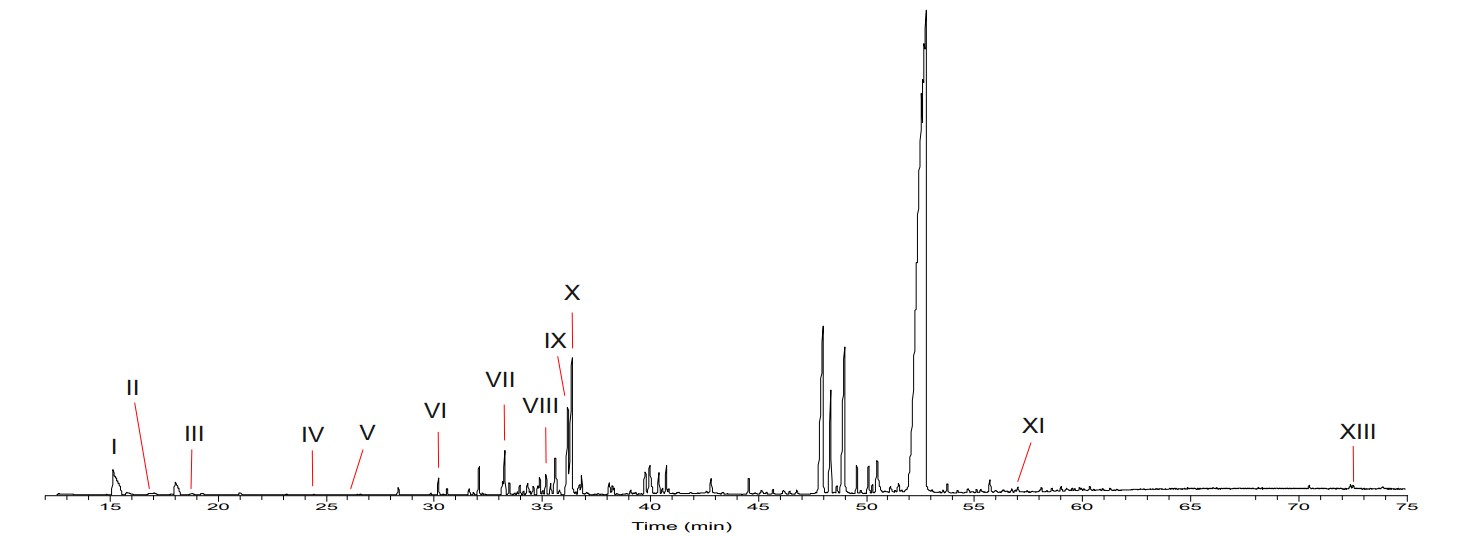


**Fig. S3 (e)** Chromatogram graph for sample A (above) and changes in chemical composition between samples A, B and C (bellow) in *Pinus sylvestris* FH


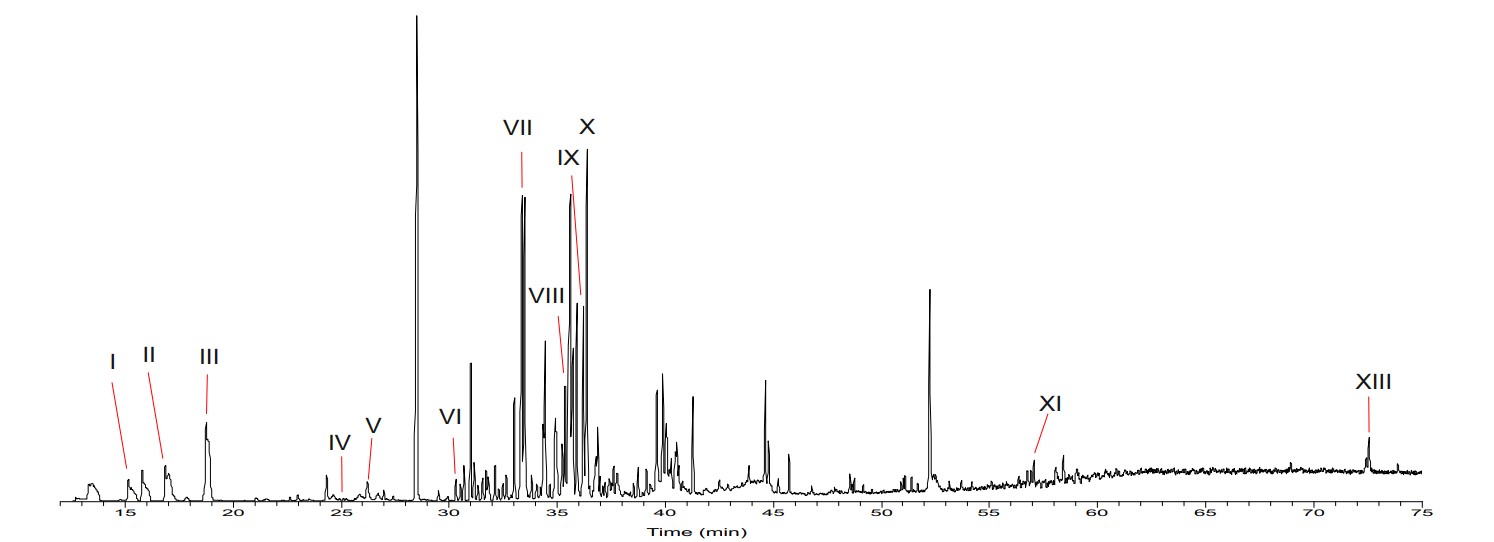


**Fig. S3 (f)** Chromatogram graph for sample A (above) and changes in chemical composition between samples A, B and C (bellow) in *Abies alba* FH


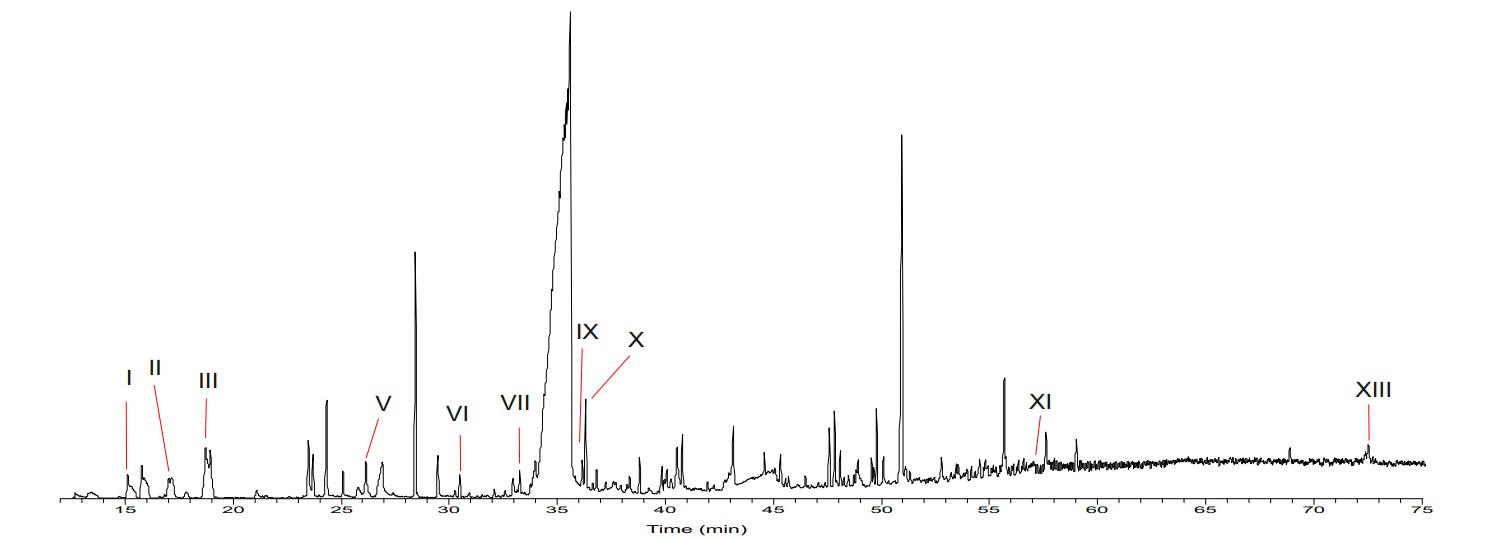


**Fig. S3 (g)** Chromatogram graph for sample A (above) and changes in chemical composition between samples A, B and C (bellow) in *Picea abies* FH

**NOTES:** **Figures S3 (a-g)** show chromatogram graphs for sample series A (n=1, free air O_3_) and changes in the chemical composition for three sample series: A (n=1, free air O_3_), B (n=3 – 3 hours of artificial ozonation), and C (n = 6 – 10 hours of artificial ozonation) analysed by CG-MS method in *Pinaceae* species needles collected in field conditions in the High Tatra Mts. region.
